# Gut microbiota pathways linking primary sclerosing cholangitis to colorectal cancer: the *Lachnospiraceae family* and PCBP1

**DOI:** 10.3389/fmicb.2026.1781475

**Published:** 2026-04-24

**Authors:** Zhaobin He, Wei Su, Zhiqiang Wang, Xiao Wang, Xiaoguang Ma, Rui Zhao

**Affiliations:** 1 Department of Hepatobiliary Surgery, Qilu Hospital of Shandong University, Jinan, Shandong, China; 2 Department of Hepatobiliary Surgery, Qinghai Red Cross Hospital, Xining, Qinghai, China

**Keywords:** colorectal cancer, Mendelian randomization, PCBP1, primary sclerosing cholangitis, the family *Lachnospiraceae*

## Abstract

**Background:**

Colorectal cancer (CRC) remains a leading cause of cancer-related death worldwide. Primary sclerosing cholangitis (PSC) increases the risk of CRC, but the biological link is not well understood. Changes in the gut-liver axis, including gut microbiota and microbial metabolites, may contribute to CRC development in PSC.

**Objective:**

To explore pathways connecting PSC with CRC through gut microbiota signals, and to identify candidate host genes for follow-up analyses.

**Methods:**

Mendelian randomization (MR) was used to estimate the causal effect of genetically predicted PSC on CRC risk and to test associations between PSC liability and gut microbial traits. Mediation analyses were performed to assess whether specific microbial features may contribute to the PSC-CRC association. Single-cell RNA sequencing (scRNA-seq) data from PSC and CRC were integrated to screen candidate host genes, which were further evaluated using survival analyses and immunohistochemistry.

**Results:**

MR supported a causal association between PSC and higher CRC risk (*OR* = 1.172, 95% *CI*: 1.033–1.329, *P* = 0.014). Genetically predicted PSC was associated with changes in gut microbial composition, with the *family Lachnospiraceae* emerging as a notable feature. Within these signals, the *genus Lachnospiraceae FCS020 group* was associated with CRC risk (*OR* = 1.237, 95% *CI*: 1.004–1.523, *P* = 0.045) and partially mediated the PSC-CRC association (mediating proportion 13.7%, 95% *CI*: 1.0%−68.1%; indirect effect 0.021, 95% *CI*: 0.000–0.054). In scRNA-seq analyses, PCBP1 emerged as a shared signal across PSC and CRC programs and was linked to butyrate related gene signatures. Higher PCBP1 expression was associated with poorer overall survival (OS) and recurrence-free survival (RFS) in CRC, and PCBP1 showed a positive association with PD-L1 by immunohistochemistry in an exploratory analysis.

**Conclusion:**

These findings provide supportive evidence that gut microbiota changes and host gene signals are linked to the PSC–CRC association. *Lachnospiraceae* related microbial features and PCBP1 warrant further validation. Studies in independent cohorts and mechanistic models will be important to clarify functional specificity and clinical utility.

## Introduction

Colorectal cancer (CRC) continues to impose a substantial global health burden, with an estimated 1.9 million new cases and 900,000 deaths each year (Wu et al., 2025). Despite advances in screening and treatment, including surgery, chemotherapy, and targeted therapies, the prognosis for patients with advanced or metastatic CRC remains poor (Singer et al., 2025). This challenge is especially clear in inflammation related CRC, where risk varies widely and the key drivers of susceptibility are still being defined. Among inflammation related risk factors, inflammatory bowel disease (IBD) is a well-established driver for CRC (Birch et al., 2022; Zhang et al., 2024). Primary sclerosing cholangitis (PSC) is a chronic cholestatic liver disease that frequently coexists with IBD and identifies a clinically distinct subgroup with markedly increased CRC risk, for whom effective surveillance and prevention remain challenging (Al Bakir et al., 2025; Eaton et al., 2020).

A central challenge is to delineate the mechanisms by which PSC amplifies CRC risk. PSC is characterized by cholestasis and altered bile acid signaling, which can affect mucosal immunity and epithelial barrier integrity, perturb the intestinal microbiota, and potentially favor colorectal carcinogenesis (Sarkar et al., 2023; Huang et al., 2025). (Blesl and Stadlbauer 2021). documented microbial alterations common to both PSC and CRC, suggesting a contributory rather than passive role in disease progression. Notably, PSC and CRC are frequently associated with gut microbial shifts characterized by reduced *Lachnospiraceae*, enrichment of *Fusobacteriaceae*, and expansion of the *phylum Proteobacteria*, often driven by increased *Enterobacteriaceae* (Hole et al., 2023; Chen et al., 2022). Microbial metabolites provide a functional bridge between these community shifts and host inflammation and barrier function (Chen et al., 2022). Among these metabolites, short-chain fatty acids (SCFAs), particularly butyrate, have drawn sustained attention because they modulate barrier integrity, immune regulation, and tumor-associated pathways in the colon (de Castilhos et al., 2024; Zhuang et al., 2025).

Butyrate-related functions offer a valuable way to study communication between the gut microbiota and the colonic mucosa. The *family Lachnospiraceae* includes many taxa that can produce butyrate, although this ability differs across lineages (Lv et al., 2024). *Lachnospiraceae* related signatures are associated with epithelial integrity and immune regulation, both central to inflammation related CRC risk (Herlo et al., 2024; Wei et al., 2021). Butyrate has anti-inflammatory and anti-tumor effects and can influence CRC through changes in gene expression, barrier support, and apoptosis in cancer cells (He et al., 2021; Chandrasekaran et al., 2025). We hypothesize that changes in *Lachnospiraceae* and butyrate-related functions contribute to PSC-associated CRC risk by altering host gene expression and immune responses, possibly through key host regulators.

This points to the need for host molecular links that connect microbial metabolites to colonic gene regulation. PCBP1 is an RNA binding protein with established roles in post transcriptional control of cellular stress responses and tumor related pathways (Yu et al., 2025). In colorectal cancer, PCBP1 has been implicated in clinically relevant phenotypes, including chemotherapy response and regulation of cell death programs, through its effects on mRNA stability and downstream gene expression networks (Shi et al., 2018; Zhu et al., 2024). These observations make PCBP1 a strong candidate host regulator. However, whether PCBP1 helps translate microbiota and metabolite changes, including shifts in *Lachnospiraceae* and butyrate related functions, into colonic transcriptional and immune alterations in the PSC setting remains unclear (Quraishi et al., 2020). We therefore investigated PCBP1 as a potential mediator linking PSC associated microbial features to host gene expression changes that may contribute to CRC progression.

**Figure 1:**
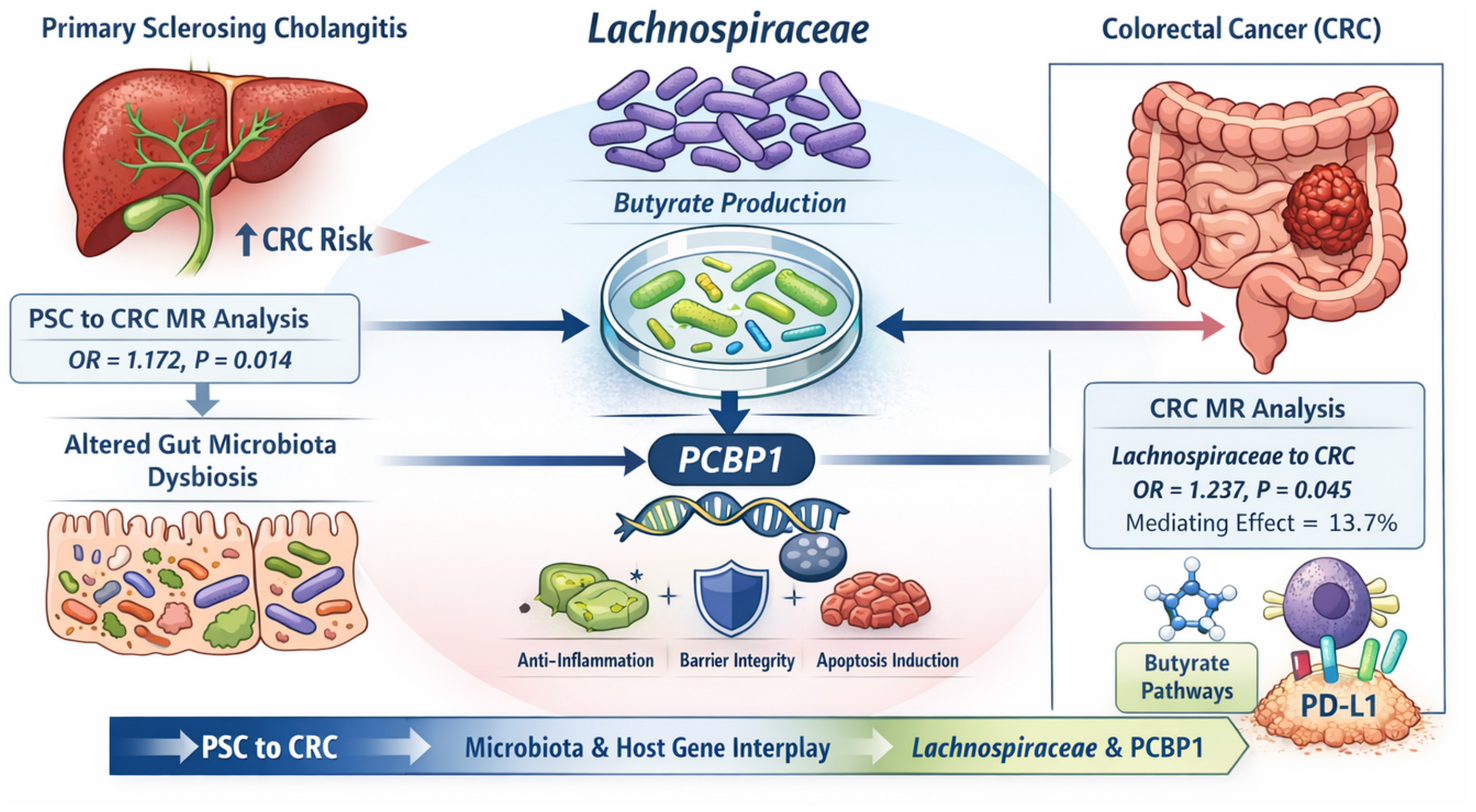
Proposed pathway linking PSC, gut microbiota, and CRC. Schematic diagram showing how PSC may be linked to CRC through changes in gut microbiota and host gene responses. Lachnospiraceae related butyrate signals and PCBP1 are highlighted as possible links in this pathway.

To address these questions, we combined Mendelian randomization (MR) with single-cell RNA sequencing (scRNA-seq). MR was used to test causal links among gut microbiota, PSC, and CRC and to clarify directionality. Single cell RNA sequencing enabled mapping of disease relevant cell types and identification of shared changes in gene expression that may connect PSC with CRC. Based on these analyses, the *family Lachnospiraceae* related butyrate signals and the host regulator PCBP1 were assessed as candidate mediators across relevant cell populations. Together, these results highlight *Lachnospiraceae*-related butyrate signals and PCBP1 as key links between PSC and CRC, providing a strong foundation for mechanistic studies (Figure 1).

## Materials and methods

### Study design

This study combined Mendelian randomization (MR), single-cell RNA sequencing (scRNA-seq), and tissue-based validation to investigate potential links between PSC, gut microbiota, and CRC. First, MR was used to test whether genetically predicted PSC is causally associated with CRC risk. MR was also used to examine causal associations between PSC liability and gut microbial traits, and between gut microbial traits and CRC risk. Mediation analyses were then performed to assess whether specific microbial features contributed to the PSC-CRC association. Next, publicly available scRNA-seq data from PSC and CRC were analyzed to identify shared gene expression patterns in relevant cell populations. These data were also used to screen candidate host genes linked to both conditions. Butyrate-related gene sets were obtained from MSigDB and used as pathway references to support gene prioritization and interpretation. Finally, immunohistochemistry was performed in our CRC tissue cohort, including tumor and paired adjacent non-tumorous tissues (Adj-NT). Inflammatory biliary specimens were additionally analyzed to provide supportive histologic evidence for PCBP1 expression.

### Ethics statement

The tissue-based part of this study was approved by the Research and Ethics Committee of Qilu Hospital, Shandong University [Approval No. KYLL-2024(ZM)-685]. Written informed consent was obtained from all participants. All analyses of publicly available GWAS and scRNA-seq data used anonymous datasets, with ethical approvals obtained in the original studies.

### Human tissue specimens and clinical data

CRC tissues and Adj-NT were collected from 80 patients who underwent curative surgical resection at Qilu Hospital, Shandong University, between July 2024 and December 2025. CRC diagnoses were independently confirmed by two gastrointestinal pathologists according to the WHO Classification of Tumors (fifth edition). Adj-NT tissues were taken >3 cm from the tumor margin, showed intact mucosa without dysplasia on histopathological review, and were further verified as tumor-free by pan-cytokeratin immunohistochemistry (AE1/AE3), when applicable.

All cases were histologically confirmed as adenocarcinoma. The cohort included 53 patients aged ≥60 years and 27 patients aged < 60 years, with 42 males and 38 females. Histopathological grading showed 68 cases with well/moderate differentiation and 12 with poor differentiation. TNM staging indicated 7 cases at stages I/II and 73 cases at stages III/IV. Molecular status was obtained from routine clinical testing when available, including 6 BRAF-positive and 74 BRAF-negative cases, as well as 31 KRAS-positive and 49 KRAS-negative cases. Clinicopathological variables were extracted from medical records and pathology reports.

Inflammatory biliary specimens were obtained from 10 patients who underwent laparoscopic cholecystectomy during the same period and were used for immunohistochemical validation. These specimens were defined as biliary tissues with histopathological confirmed inflammation, including lymphoplasmacytic and/or neutrophilic infiltrates, mucosal injury/edema and/or fibrosis, and no evidence of malignancy. For each case, the inflammatory diagnosis was established by routine pathology review, and representative FFPE blocks were selected for PCBP1 staining.

### Public data sources

Genetic instruments for gut microbiota traits were obtained from the MiBioGen consortium (https://mibiogen.gcc.rug.nl/), which analyzed genome-wide genotypes and 16S rRNA fecal microbiome profiles from 18,340 individuals across 24 cohorts. Summary statistics were adjusted for age, sex, technical covariates, and genetic principal components.

PSC summary statistics were obtained from FinnGen (Version 8; https://mibiogen.gcc.rug.nl/menu/main/home/). CRC GWAS summary statistics were derived from (Liberzon et al. 2015), as indexed in the GWAS Catalog (https://www.ebi.ac.uk/gwas/).

Gene sets related to butyrate were retrieved from the Molecular Signatures Database (MSigDB; https://www.gsea-msigdb.org/gsea/msigdb/index.). Since MSigDB annotations often use the term “butyric acid,” we used this term as the query but refer to these as butyrate-related genes throughout the manuscript for terminological consistency.

CRC scRNA-seq data (GSE261388) and PSC scRNA-seq data (GSE247128) were downloaded from the Gene Expression Omnibus (GEO; https://www.ncbi.nlm.nih.gov/geo/).

### Immunohistochemistry (IHC)

PCBP1 protein expression was evaluated on 3-μm formalin-fixed paraffin-embedded (FFPE) sections. After deparaffinization and rehydration, antigen retrieval was performed in citrate buffer (pH 6.0) using microwave heating. Endogenous peroxidase activity was blocked with 3% hydrogen peroxide for 10 min. Sections were incubated overnight at 4 °C with a rabbit anti-PCBP1 primary antibody (1:100; Santa Cruz Biotechnology), followed by an HRP-conjugated goat anti-rabbit secondary antibody (1:500; Santa Cruz Biotechnology) for 2 h at room temperature. Signals were visualized using 3,3′-diaminobenzidine (DAB), and nuclei were counterstained with hematoxylin. Negative controls were processed in parallel with omission of the primary antibody.

Staining was quantified using the *H*-score system: *H*-score = 1 × (% weak) + 2 × (% moderate) + 3 × (% strong), yielding a range of 0–300. Five randomly selected high-power fields ( × 400; excluding necrotic areas) were scored per slide by two observers blinded to group labels; disagreements were resolved by consensus.

## Mendelian randomization analysis

### Instrument selection and harmonization

For PSC as exposure, SNPs associated at genome-wide significance (*P* < 5 × 10^−8^) were clumped to ensure independence (*R*^2^ < 0.001, 10,000 kb window). For each microbial taxon, SNPs associated at *P* < 1.0 × 10^−5^ were used as instruments. In reverse-direction analyses, SNPs associated with colon cancer at *P* < 5 × 10^−8^ served as instruments. Exposure and outcome datasets were harmonized to align effect alleles, and palindromic SNPs with ambiguous strand alignment were excluded. Instrument strength was evaluated using the *F* statistic, with *F* > 10 indicating adequate strength.

After excluding unnamed microbial traits, 196 microbial traits were retained (9 phyla, 16 classes, 20 orders, 32 families, and 119 genera). In MiBioGen, some genus-level microbial traits are annotated as unclassified clades within a higher-rank lineage and are reported using placeholder labels such as “UCG” or “group” (for example, *Lachnospiraceae FCS020 group, Lachnospiraceae UCG004 group, Lachnospiraceae UCG008 group, and Eubacterium hallii group*). In addition, genus-level features may occasionally retain higher-rank lineage names when genus assignment is unresolved (for example, *genus-level Christensenellaceae clade*). We therefore report taxa using the original MiBioGen trait names, and such labels represent genus-level clades within the corresponding bacterial lineage.

### Primary MR estimation and sensitivity analyses

Two-sample MR was conducted using inverse-variance weighted (IVW) analysis as the primary estimator, with weighted median and MR-Egger regression as complementary methods. Heterogeneity across SNP-specific estimates was assessed using Cochran's *Q*-test, and random-effects IVW was applied when heterogeneity was detected (*P* < 0.05). Horizontal pleiotropy was evaluated using the MR-Egger intercept, and outliers were assessed using MR-PRESSO. Leave-one-out analyses were performed to examine the influence of individual variants.

Statistical significance was assessed primarily using the IVW estimate. For microbiome-wide analyses across taxa, *P* values were adjusted using the Benjamini–Hochberg false discovery rate (FDR) method, with *q* < 0.1 considered statistically significant.

### Mediation analysis

Mediation was evaluated under standard MR assumptions, and was considered valid when a directionally consistent pathway was observed: PSC influenced the mediator, and the mediator influenced CRC. Directionality was assessed using bidirectional MR. Consistency across MR methods (IVW, weighted median, MR-Egger) was assessed, along with the absence of heterogeneity, pleiotropy, or influential variants, using Cochran's Q, MR-Egger intercept, MR-PRESSO, and leave-one-out analyses. Only consistent findings across all tests were considered robust evidence for mediation.

All MR analyses were performed in R using the packages Two Sample MR, Mendelian Randomization, and MR-PRESSO.

### Single-cell RNA-seq processing and overlap analysis

scRNA-seq data were processed in R using Seurat. After standard quality control and normalization, highly variable genes (top 2,000) were identified. Principal component analysis (PCA) was performed, and the number of principal components used for downstream analyses was determined using ElbowPlot and PC heatmaps. Cells were clustered using a shared nearest-neighbor approach (FindNeighbors/FindClusters) and visualized using UMAP. Cluster marker genes were identified using the Wilcoxon rank-sum test with Benjamini–Hochberg correction; markers were defined as genes with adjusted *P* < 0.05 and |log fold change| > 0.25. Cell types and subclusters were annotated based on canonical marker expression.

To identify candidate genes linking CRC, PSC, and butyrate-related pathways, we intersected epithelial cell–specific genes from CRC, cholangiocyte-specific genes from PSC, and butyrate-related genes (MSigDB). Venn diagrams were generated in R, and intersection sets were extracted programmatically for downstream analyses.

### Statistical analysis

Statistical analyses were performed in R (version 4.3.1) and GraphPad Prism 8.0 (GraphPad Software, Inc.). Continuous variables are summarized as medians, and categorical variables as counts and percentages. Survival analyses were conducted using Kaplan–Meier curves with log-rank tests. Cox proportional hazards models were used to estimate hazard ratios (HRs) with 95% confidence intervals (95% CIs). All tests were two-tailed, and *P* < 0.05 was considered statistically significant unless otherwise specified. For microbiome-wide MR screening, FDR-adjusted q-values were used as described above.

## Results

### Genetically predicted PSC is associated with higher CRC risk

**Figure 2:**
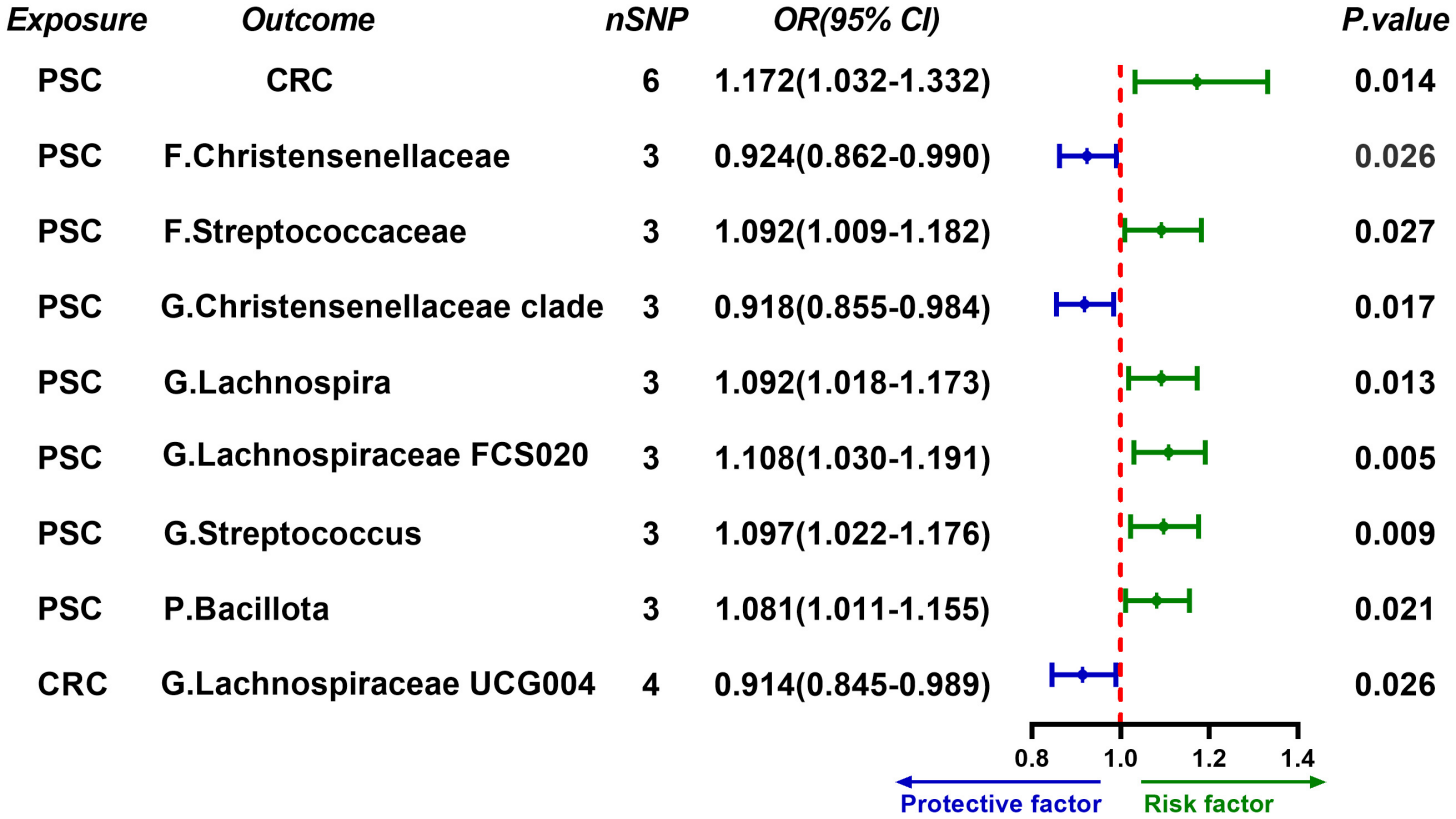
Forward MR results from PSC to CRC risk and gut microbial traits. Forest plot shows ORs and 95% CIs for the associations of genetically predicted PSC with CRC risk and with gut microbial traits. The dashed vertical line indicates *OR* = 1. Green indicates risk direction (*OR* > 1) and blue indicates protective direction (*OR* < 1). nSNP denotes the number of instrumental variants; F., G., and P. indicate family-, genus-, and phylum-level traits, respectively. MR, Mendelian randomization; OR, odds ratio; CI, confidence interval.

Genetic analysis identified 2,607 SNPs significantly associated with PSC (*P* < 5 × 10^−8^). After linkage disequilibrium (LD) clumping (*R*^2^ < 0.001; Clumping distance = 10,000 kb), 6 independent SNPs were retained as instrumental variables (Supplementary Table S1). Two-sample MR analysis supported a causal effect of PSC on CRC risk, with an OR of 1.172 (95% *CI*: 1.032–1.332, *P* = 0.014) (Figure 2).

### Genetically predicted PSC is associated with shifts in gut microbial traits

PSC was associated with increased abundance of five taxa, including the *family Streptococcaceae* (*OR* = 1.092, 95% *CI*: 1.009–1.182, *P* = 0.044), the *genus Lachnospira* (*OR* = 1.092, 95% *CI*: 1.018–1.173, *P* = 0.013), the *genus Lachnospiraceae FCS020 group* (*OR* = 1.108, 95% *CI*: 1.030–1.191, *P* = 0.005), the *genus Streptococcus* (*OR* = 1.097, 95% *CI*: 1.022–1.176, *P* = 0.009), and the *phylum Bacillota* (*OR* = 1.081, 95% *CI*: 1.011–1.155, *P* = 0.021). Conversely, PSC was associated with decreased abundance of two taxa, including the *family Christensenellaceae* (*OR* = 0.924, 95% *CI*: 0.862–0.990, *P* = 0.026) and the *genus-level Christensenellaceae clade* (*OR* = 0.918, 95% *CI*: 0.855–0.948, *P* = 0.017) (Figure 2 and Supplementary Table S2).

### Gut microbial traits show causal associations with CRC risk

**Figure 3:**
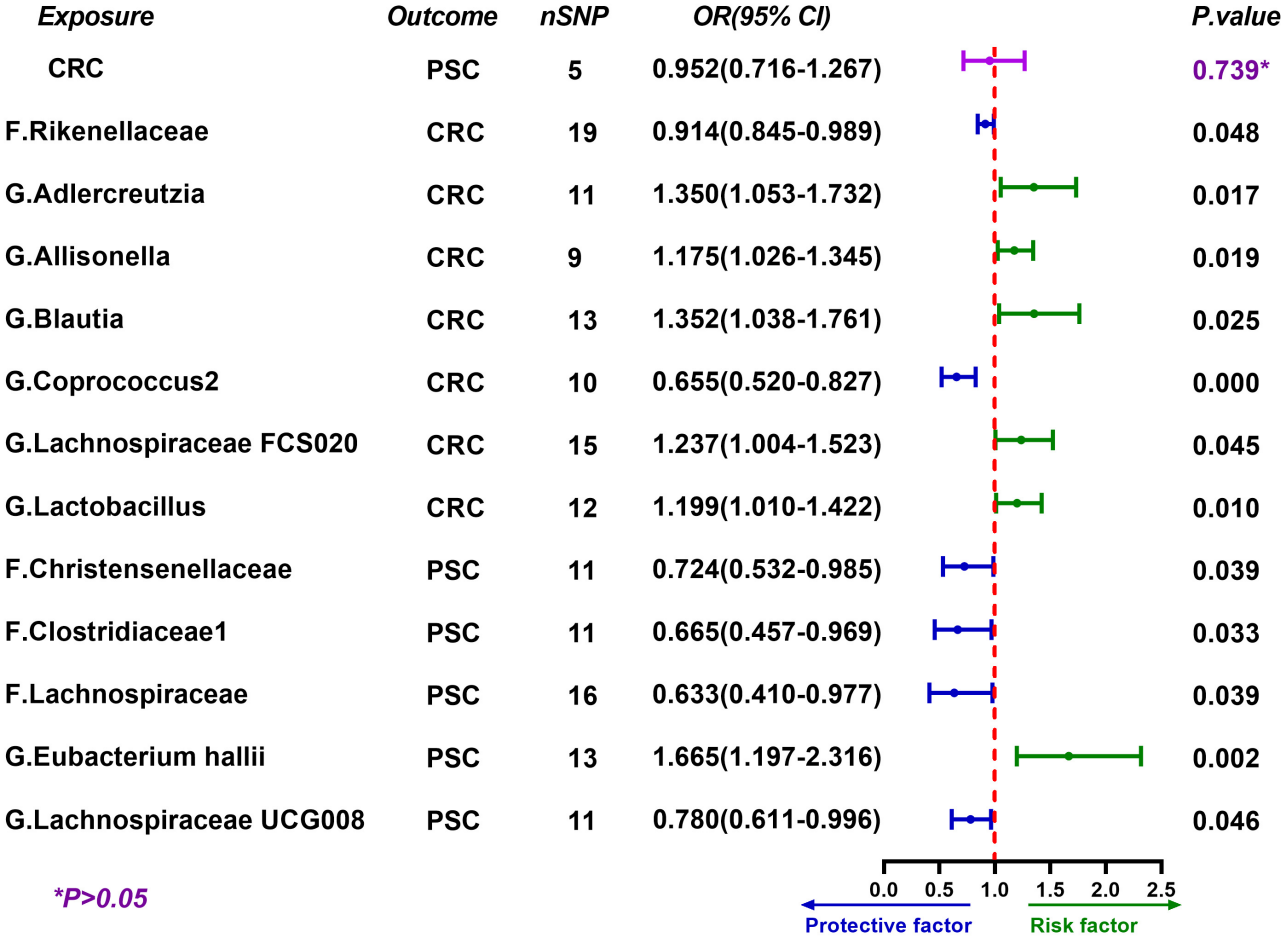
Bidirectional MR results for gut microbial traits, CRC, and PSC. Forest plot shows ORs and 95% CIs for the associations of gut microbial traits with CRC risk and with PSC, together with the reverse-direction estimate for CRC on PSC. The dashed vertical line indicates *OR* = 1. Green indicates risk direction (*OR* > 1) and blue indicates protective direction (*OR* < 1). nSNP denotes the number of instrumental variants; F. and G. indicate family- and genus-level traits, respectively. MR, Mendelian randomization; OR, odds ratio; CI, confidence interval.

When evaluating the causal associations between gut microbial traits and CRC risk, seven taxa showed significant associations. The *genus Coprococcus2* was associated with reduced CRC risk (*OR* = 0.655, 95% *CI*: 0.520–0.827, *P* = 3.0 × 10^−4^). In contrast, six taxa were associated with increased CRC risk, including the *family Rikenellaceae* (*OR* = 1.243, 95% *CI*: 1.001–1.542, *P* = 0.048), the *genus Adlercreutzia* (*OR* = 1.350, 95% *CI*: 1.053–1.732, *P* = 0.017), the *genus Allisonella* (*OR* = 1.175, 95% *CI*: 1.026–1.345, *P* = 0.019), the *genus Blautia* (*OR* = 1.352, 95% *CI*: 1.038–1.761, *P* = 0.025), the *genus Lachnospiraceae FCS020 group* (*OR* = 1.237, 95% *CI*: 1.004–1.523, *P* = 0.045), and the *genus Lactobacillus* (*OR* = 1.199, 95% *CI*: 1.010–1.422, *P* = 0.010) (Figure 3 and Supplementary Table S2).

In reverse MR analysis, CRC showed no evidence of a causal effect on PSC (*OR* = 0.952, 95% *CI*: 0.716–1.267, *P* = 0.739). However, CRC exhibited a causal effect on *genus Lachnospiraceae UCG004 group* (*OR* = 0.914, 95% *CI*: 0.845–0.989, *P* = 0.026) (Figure 2). Moreover, several gut microbiota taxa showed causal effects on PSC, including the *family Christensenellaceae* (*OR* = 0.724, 95% *CI*: 0.532–0.985, *P* = 0.039), the *family Clostridiaceae1* (*OR* = 0.665, 95% *CI*: 0.457–0.969, *P* = 0.033), the *family Lachnospiraceae* (*OR* = 0.633, 95% *CI*: 0.410–0.977, *P* = 0.039), the *genus Eubacterium hallii group* (*OR* = 1.665, 95% *CI*: 1.197–2.316, *P* = 0.002) and the *genus Lachnospiraceae UCG008 group* (*OR* = 0.780, 95% *CI*: 0.611–0.996, *P* = 0.046) (Figure 3).

### *Genus Lachnospiraceae FCS020 group* partially mediates the PSC–CRC association

Our findings suggest that the *genus Lachnospiraceae FCS020 group* partially mediates the PSC–CRC association. Genetically predicted PSC was associated with the *Lachnospiraceae FCS020 group* (*OR* = 1.108, 95% *CI*: 1.030–1.191, *P* = 0.005), and the *Lachnospiraceae FCS020 group* showed a causal effect on CRC (*OR* = 1.237, 95% *CI*: 1.004–1.523, *P* = 0.045). The mediation analysis yielded an indirect effect of 0.021 (95% *CI*: 0.000–0.054), accounting for 13.7% (95% *CI*: 1.0%−68.1%) of the total effect, supporting a mediating role of this genus-level clade in the PSC–CRC association.

To ensure the robustness of our findings, we performed sensitivity analyses. MR-Egger regression provided no evidence of directional pleiotropy, and MR-PRESSO detected no horizontal pleiotropy (global test *P* > 0.05; Supplementary Table S3). Cochran's Q test indicated no significant heterogeneity (*P* > 0.05; Supplementary Table S3). Leave-one-out analyses further confirmed that the overall estimates were not driven by any single SNP (Supplementary Figures S1–S13).

### Identification of PCBP1 across CRC, PSC, and butyrate-related genes

**Figure 4:**
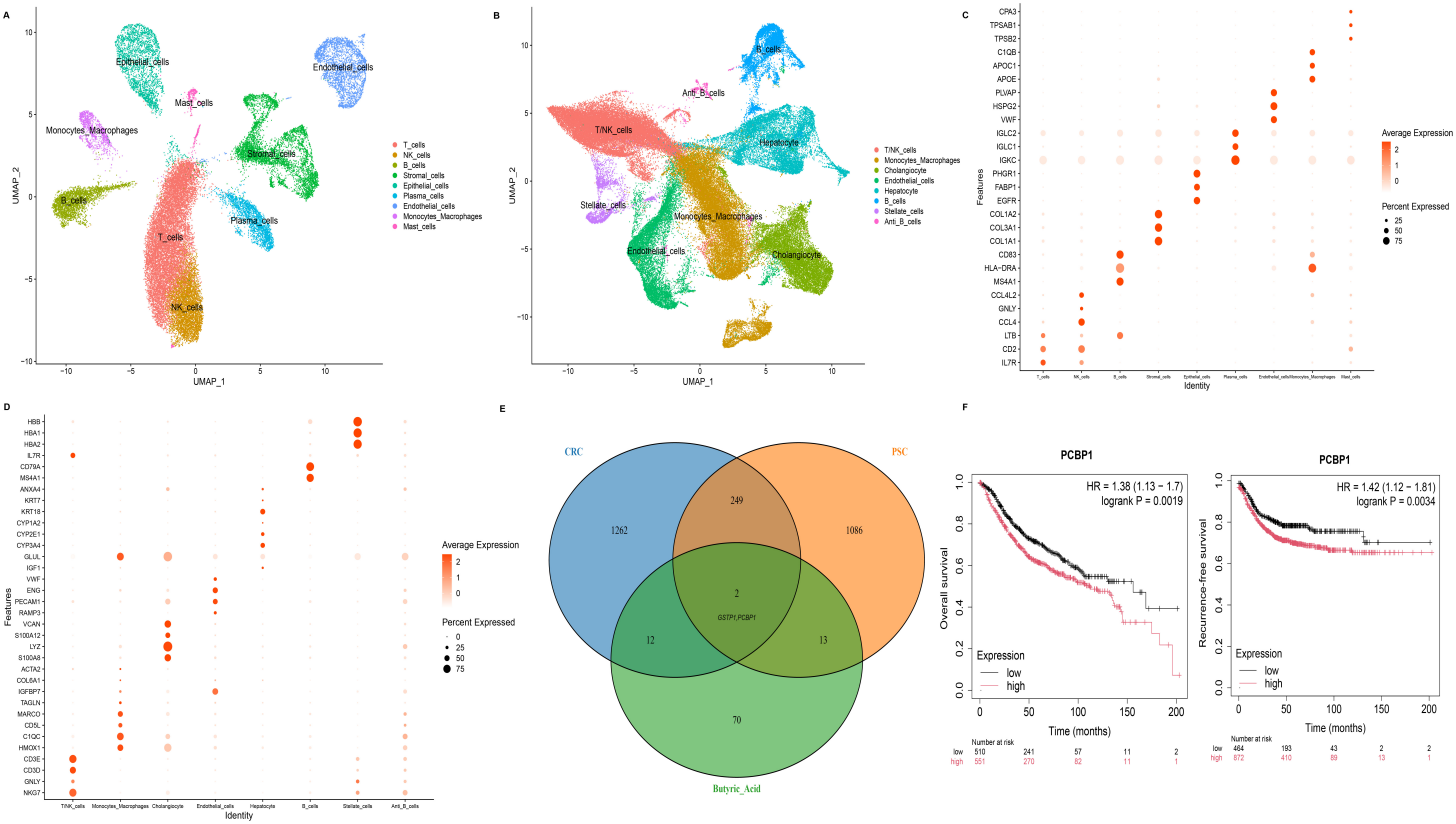
Integrated single-cell and survival analyses identify PCBP1 as a shared signal across CRC, PSC, and butyrate-related signatures. **(A)** UMAP plot showing major cell populations in CRC tissue. **(B)** UMAP plot showing major cell populations in PSC tissue. **(C)** Dot plot displaying the top differentially expressed genes in CRC samples. **(D)** Dot plot displaying the top differentially expressed genes in PSC samples. **(E)** Venn diagram showing the overlap among CRC epithelial cell-specific genes, PSC cholangiocyte-specific genes, and butyrate-related signatures. **(F)** Kaplan–Meier survival curves showing the association of PCBP1 expression with overall survival and recurrence-free survival in CRC. UMAP, uniform manifold approximation and projection; scRNA-seq, single-cell RNA sequencing; CRC, colorectal cancer; PSC, primary sclerosing cholangitis.

scRNA-seq analyses of CRC and PSC datasets revealed distinct cellular landscapes. In the CRC dataset, epithelial cells, T cells, NK cells, B cells, stromal cells, plasma cells, endothelial cells, monocytes/macrophages, and mast cells were identified (Figure 4A). In the PSC dataset, major cell populations included T/NK cells, monocytes/macrophages, cholangiocytes, endothelial cells, hepatocytes, B cells, and stellate cells (Figure 4B). Differential expression analyses identified characteristic marker genes for CRC epithelial cells and PSC cholangiocytes, supporting the accuracy of cell-type annotation (Figures 4C, D).

To explore potential molecular links between CRC, PSC, and butyrate-related pathways, we performed an intersection analysis of epithelial cell–specific genes from CRC (*n* = 1,262), cholangiocyte-specific genes from PSC (*n* = 1,086), and butyrate-related genes (*n* = 701). Our Venn diagram analysis revealed limited overlap among the three gene sets, with only two genes (GSTP1 and PCBP1) shared across all three conditions (Figure 4E). In survival analyses of CRC patients, higher PCBP1 expression was significantly associated with poorer overall survival (OS; *HR* = 1.38, *P* = 0.002) and recurrence-free survival (RFS; *HR* = 1.42, *P* = 0.003) in CRC patients (Figure 4F), supporting PCBP1 as a candidate gene linking cellular programs in PSC and CRC.

### PCBP1 expression in CRC and inflammatory biliary tissues

**Figure 5:**
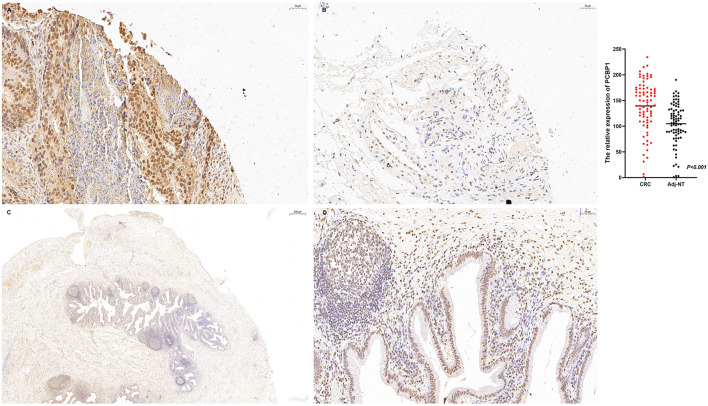
Tissue validation of PCBP1 expression in CRC and inflammatory biliary tissues. IHC images show PCBP1 staining in CRC tissue **(A)** and adjacent non-tumor tissue **(B)**. Quantification by *H*-score showed higher PCBP1 expression in CRC than in adjacent non-tumor tissues (139.34 ± 49.63 vs. 104.76 ± 40.41, *P* < 0.001). PCBP1 immunoreactivity is also shown in inflammatory biliary tissue at low magnification (**C**; scale bar 500 μm) and high magnification (**D**; scale bar 50 μm). IHC, immunohistochemistry; CRC, colorectal cancer; Adj-NT, adjacent non-tumor tissue.

IHC further confirmed PCBP1 expression in CRC tissues and inflammatory biliary specimens. In CRC tissues, PCBP1 was predominantly localized to the cytoplasm, and tumor samples exhibited significantly higher *H*-scores than Adj-NT tissues (*P* < 0.001; Figures 5A, B). PCBP1 immunoreactivity was also detectable in inflammatory biliary tissues, supporting its involvement in inflammatory biliary contexts (Figures 5C, D).

To examine potential immune relevance, we assessed correlations between PCBP1 and immune markers. PCBP1 expression showed a positive correlation with PD-L1 (Pearson's *r* = 0.29, *P* = 0.01), whereas no significant correlation was observed with CD8 (Pearson's *r* = −0.12, *P* = 0.29), suggesting a possible association with immune regulatory signaling rather than cytotoxic T-cell infiltration in these samples.

## Discussion

In this study, we investigated the causal relationship between primary PSC and CRC using MR and explored the role of gut microbiota in this association. Genetically predicted PSC was associated with an increased risk of CRC. We also observed that PSC was linked to changes in gut microbial composition across various taxonomic ranks, including the *families Rhodospirillaceae* and *Streptococcaceae*, and the *genus Streptococcus*. Among these microbial signals, the *genus Lachnospiraceae FCS020 group* consistently emerged as a key mediator, showing associations in both the PSC-to-microbiota and microbiota-to-CRC analyses. Mediation analysis suggested that this genus-level clade accounted for approximately 13.7% of the total effect. Furthermore, we identified PCBP1 as a shared molecular signal across CRC epithelial programs, PSC cholangiocyte programs, and butyrate-related gene sets, suggesting a potential host regulatory link between these conditions. Notably, PCBP1 was also associated with overall survival and recurrence-free survival in CRC, emphasizing its clinical relevance.

PSC is increasingly recognized as a cholestatic disease often accompanied by IBD, particularly ulcerative colitis (UC) (Yasuda et al., 2025). Previous studies have reported that up to 80% of patients with PSC have concurrent IBD, and that 2% to 7.5% of patients with IBD develop PSC (Punia et al., 2021; Li et al., 2025; van Munster et al., 2024). This overlap is clinically meaningful because PSC with IBD differs from other forms of IBD in clinical characteristics and cancer risk, particularly CRC (Lavelle et al., 2022). Our MR findings support a causal association between PSC and CRC risk, providing a rationale for further investigation into mechanisms and prevention strategies in this high-risk population.

The relationship between PSC and the gut microbiota has gained increasing attention because it may influence both PSC progression and CRC risk. Altered bile acid metabolism is a key feature of PSC and can change microbial survival and community structure in the gastrointestinal tract. Microbial shifts in the gut, including the upper gastrointestinal tract and colon, may play a role in both PSC and CRC development (Toribio-Mateas et al., 2021). Studies comparing the microbiota of PSC patients with healthy controls have consistently shown significant differences. For example, Bajer et al. (Bajer et al., 2017) found higher levels of taxa such as the genus *Rothia, Enterococcus, Streptococcus, and Veillonella* in PSC, mostly independent of IBD. Our MR analyses also suggested that genetically predicted PSC is linked to genus *Lachnospira and Streptococcus*-related signals, indicating a possible causal connection between PSC and certain microbial features, rather than just an observational relationship. Additionally, (Cui et al. 2024) found that the *families Clostridiaceae1 and Lachnospiraceae* were linked to a lower risk of PSC, suggesting that *Lachnospiraceae* may have a protective role in some cases. Consistent with this, our reverse-direction MR analysis showed a similar protective association for the *family Clostridiaceae1 and Lachnospiraceae*. Together, these findings suggest that the role of *Lachnospiraceae*-related signals may vary depending on the context, highlighting the need for further research into how microbial diversity and related metabolites contribute to PSC-associated CRC.

An important part of the connection between the microbiota and CRC involves microbial metabolites, especially butyrate, a short-chain fatty acid produced by some gut bacteria, including members of the *Lachnospiraceae family* (Su et al., 2023; Baas et al., 2024; Yang et al., 2025; Zhang et al., 2025). Butyrate has been shown to have anti-inflammatory and tumor-suppressive effects in the colon by regulating gene expression and promoting apoptosis (Mohammadi et al., 2023; Wu et al., 2021; Ruiz-Álvarez et al., 2025). It reduces inflammation in both epithelial and immune cells by lowering NF-κB activity and can influence growth and stress response pathways like PI3K/AKT/mTOR, which supports the link between butyrate and CRC (Tong et al., 2022; Cheng et al., 2022). In our MR analyses, the *genus Lachnospiraceae FCS020 group* was positively linked to CRC risk, which may seem at odds with the idea that Lachnospiraceae is always beneficial. This could be explained by the diversity within the Lachnospiraceae family, as not all species produce butyrate. According to (Awoniyi et al. 2023), only 40.8% of genomes in this family encode a full butyrate synthesis pathway. Furthermore, PSC involves changes in bile acids and ongoing inflammation, which can alter the gut microbiota and promote bacteria that thrive in an inflammatory environment (Quraishi et al., 2025). Therefore, the observed effects should not be attributed to *Lachnospiraceae* as a whole but need to be examined at a more specific level. Our results suggest that the *genus Lachnospiraceae FCS020 group* could mediate part of the PSC to CRC link. However, since butyrate was not directly measured in this study, our evidence is based on gene expression data related to butyrate rather than its actual presence in the gut. These findings should be viewed as potential leads, and further validation with metabolomics and microbiome profiling in PSC and CRC patients is needed.

Poly(C)-binding protein 1, also known as hnRNP E1, is a widely expressed RNA binding protein in the heterogeneous nuclear ribonucleoprotein family. PCBP1 contains three K homology domains and binds cytosine rich sequences in target messenger RNAs. It participates in transcriptional and post transcriptional regulation and can affect messenger RNA stability, translation, and alternative splicing (Wang and Yang, 2021; Liu et al., 2023). PCBP1 also serves as an iron chaperone and contributes to cellular iron homeostasis by supporting iron storage and use (Majerníková et al., 2024). Recent studies suggest that PCBP1 can play different roles across cancer types and microenvironments. For example, (Zhang P. et al. 2025) reported in gastric cancer that circular RNAs can modulate PCBP1 through post translational changes and may contribute to tumor progression under hypoxia. In colorectal cancer, (Yu et al. 2025) identified PCBP1 as a target of acevaltrate and linked PCBP1 to iron metabolism, which supports interest in PCBP1 in relation to ferroptosis based strategies.

In our study, we examined PCBP1 by using an intersection analysis of genes from PSC, CRC, and butyrate related gene sets. This analysis nominated PCBP1 as a shared signal across these data layers. We further found that higher PCBP1 expression was associated with poorer overall survival and recurrence free survival in CRC, which supports its potential clinical relevance. In tissue-based analyses, PCBP1 staining was detectable, and PCBP1 showed a positive association with PD-L1. This point is relevant given the growing interest in microbiota effects on immune checkpoint responses and immune related adverse events. While our data are correlative, they support future work to test whether the PSC related microbiota context and PCBP1 linked programs shape checkpoint related immune states in CRC. This finding suggests that PCBP1 may relate to immune regulatory states, although the current data support association rather than direct regulation. Together, these results support PCBP1 as a candidate biomarker and a plausible host node linking inflammatory context, iron handling, and cancer related programs. Future work should focus on clarifying upstream drivers and downstream pathways of PCBP1, and on validating its utility for patient stratification and therapeutic development in PSC associated CRC.

Several limitations should be considered when interpreting our findings. First, butyrate was not directly measured in this study. The butyrate related component was based on curated gene sets, which reflect transcriptional programs rather than metabolite exposure. Future studies should include targeted measurement of short chain fatty acids and functional microbiome profiling to validate this mechanism. Second, our immunohistochemistry validation used CRC tissues and institutional inflammatory biliary specimens, and the number of inflammatory biliary specimens was limited to 10. These data provide supportive evidence, but they limit generalizability and make the immune marker correlations exploratory. Larger independent cohorts, ideally including well characterized PSC tissues, will be needed to confirm PCBP1 expression patterns and immune associations. Third, although MR can reduce confounding and reverse causation, it may still be affected by pleiotropy, heterogeneity, and limited instrument strength for some microbial traits. We used multiple sensitivity analyses and adjusted for multiple testing, but replication in independent datasets and functional experiments remains important. Overall, future work that combines metabolomics, longitudinal cohorts, and experimental models will help validate the proposed pathway, refine microbial functional contributors at finer taxonomic resolution, and clarify how PCBP1 relates to immune and metabolic regulation in this setting.

## Conclusion

Our study clarifies a gut microbiota and host gene pathway that links PSC to CRC. MR supports a causal association between PSC and a higher risk of CRC. Among PSC related microbial changes, the *genus Lachnospiraceae FCS020 group* showed a consistent association with CRC risk. It also partially mediated the link between PSC and CRC. In parallel, PCBP1 emerged as a shared signal in PSC and CRC single cell analyses and in butyrate related gene programs. Higher PCBP1 expression was also associated with worse CRC outcomes. Together, these results highlight the *Lachnospiraceae family* and PCBP1 as priority candidates for risk stratification and mechanism-based intervention in PSC associated CRC. These findings still require validation in independent cohorts and confirmation in functional studies.

## Data Availability

The datasets presented in this study can be found in online repositories. The names of the repository/repositories and accession number(s) can be found in the article/Supplementary material.

## References

[B1] Al Bakir I. Curtius K. Cresswell G. D. Grant H. E. Nasreddin N. Smith K. . (2025). Low-coverage whole genome sequencing of low-grade dysplasia strongly predicts advanced neoplasia risk in ulcerative colitis. Gut 74, 740–751. doi: 10.1136/gutjnl-2024-333353 39880602 PMC12013573

[B2] Awoniyi M. Wang J. Ngo B. Meadows V. Tam J. Viswanathan A. . (2023). Protective and aggressive bacterial subsets and metabolites modify hepatobiliary inflammation and fibrosis in a murine model of PSC. Gut 72, 671–685. doi: 10.1136/gutjnl-2021-326500 35705368 PMC9751228

[B3] Baas F. S. Brusselaers N. Nagtegaal I. D. Engstrand L. Boleij A. (2024). Navigating beyond associations: opportunities to establish causal relationships between the gut microbiome and colorectal carcinogenesis. Cell Host Microbe 32, 1235–1247. doi: 10.1016/j.chom.2024.07.008 39146796

[B4] Bajer L. Kverka M. Kostovcik M. Macinga P. Dvorak J. Stehlikova Z. . (2017). Distinct gut microbiota profiles in patients with primary sclerosing cholangitis and ulcerative colitis. World J. Gastroenterol. 23, 4548–4558. doi: 10.3748/wjg.v23.i25.4548 28740343 PMC5504370

[B5] Birch R. J. Burr N. Subramanian V. Tiernan J. P. Hull M. A. Finan P. . (2022). Inflammatory bowel disease-associated colorectal cancer epidemiology and outcomes: an English population-based study. Am. J. Gastroenterol. 117, 1858–1870. doi: 10.14309/ajg.0000000000001941 36327438

[B6] Blesl A. Stadlbauer V. (2021). The gut-liver axis in cholestatic liver diseases. Nutrients 13:1018. doi: 10.3390/nu13031018 33801133 PMC8004151

[B7] Chandrasekaran P. Krausz M. Han Y. Mitsuiki N. Gabrysch A. Nöltner C. . (2025). The intestinal microbiome and metabolome discern disease severity in cytotoxic T-lymphocyte-associated protein 4 deficiency. Microbiome 13:51. doi: 10.1186/s40168-025-02028-7 39934899 PMC11817180

[B8] Chen F. Dai X. Zhou C. C. Li K. X. Zhang Y. J. Lou X. Y. . (2022). Integrated analysis of the faecal metagenome and serum metabolome reveals the role of gut microbiome-associated metabolites in the detection of colorectal cancer and adenoma. Gut 71, 1315–1325. doi: 10.1136/gutjnl-2020-323476 34462336 PMC9185821

[B9] Cheng X. Tan Y. Li H. Huang J. Zhao D. Zhang Z. . (2022). Fecal 16S rRNA sequencing and multi-compartment metabolomics revealed gut microbiota and metabolites interactions in APP/PS1 mice. Comput. Biol. Med. 151(Pt A):106312. doi: 10.1016/j.compbiomed.2022.106312 36417828

[B10] Cui Y. Guo Y. Kong Y. Zhang G. (2024). Association between gut microbiota and autoimmune cholestatic liver disease, a Mendelian randomization study. Front. Microbiol. 15:1348027. doi: 10.3389/fmicb.2024.1348027 38601930 PMC11004368

[B11] de Castilhos J. Tillmanns K. Blessing J. Laraño A. Borisov V. Stein-Thoeringer C. K. (2024). Microbiome and pancreatic cancer: time to think about chemotherapy. Gut Microbes 16:2374596. doi: 10.1080/19490976.2024.2374596 39024520 PMC11259062

[B12] Eaton J. E. Vesterhus M. McCauley B. M. Atkinson E. J. Schlicht E. M. Juran B. D. . (2020). Primary sclerosing cholangitis risk estimate tool (PREsTo) predicts outcomes of the disease: a derivation and validation study using machine learning. Hepatology 71, 214–224. doi: 10.1002/hep.30085 29742811 PMC6226358

[B13] He Y. Sun M. M. Zhang G. G. Yang J. Chen K. S. Xu W. W. . (2021). Targeting PI3K/Akt signal transduction for cancer therapy. Signal Transduct. Target Ther. 6:425. doi: 10.1038/s41392-021-00828-5 34916492 PMC8677728

[B14] Herlo L. F. Salcudean A. Sirli R. Iurciuc S. Herlo A. Nelson-Twakor A. . (2024). Gut microbiota signatures in colorectal cancer as a potential diagnostic biomarker in the future: a systematic review. Int. J. Mol. Sci. 25:7937. doi: 10.3390/ijms25147937 39063179 PMC11276678

[B15] Hole M. J. Jørgensen K. K. Holm K. Braadland P. R. Meyer-Myklestad M. H. Medhus A. W. . (2023). A shared mucosal gut microbiota signature in primary sclerosing cholangitis before and after liver transplantation. Hepatology 77, 715–728. doi: 10.1002/hep.32773 36056902 PMC9936983

[B16] Huang M. Huang M. Liu L. Yang F. He C. Sun Y. C. . (2025). Gut microbiota modulates obesity-associated skeletal deterioration through macrophage aging and grancalcin secretion. Adv. Sci. (Weinh.). 12:e2502634. doi: 10.1002/advs.202502634 40349163 PMC12302596

[B17] Lavelle A. Nancey S. Reimund J. M. Laharie D. Marteau P. Treton X. . (2022). Fecal microbiota and bile acids in IBD patients undergoing screening for colorectal cancer. Gut Microbes 14:2078620. doi: 10.1080/19490976.2022.2078620 35638103 PMC9176255

[B18] Li L. Li T. Liang X. Zhu L. Fang Y. Dong L. . (2025). A decrease in flavonifractor plautii and its product, phytosphingosine, predisposes individuals with phlegm-dampness constitution to metabolic disorders. Cell Discov. 11:25. doi: 10.1038/s41421-025-00789-x 40097405 PMC11914097

[B19] Liberzon A. Birger C. Thorvaldsdóttir H. Ghandi M. Mesirov J. P. Tamayo P. (2015). The molecular signatures database (MSigDB) hallmark gene set collection. Cell Syst. 1, 417–425. doi: 10.1016/j.cels.2015.12.004 26771021 PMC4707969

[B20] Liu Y. Peng L. Chen J. Chen L. Wu Y. Cheng M. . (2023). EIF5A2 specifically regulates the transcription of aging-related genes in human neuroblastoma cells. BMC Geriatr. 23:83. doi: 10.1186/s12877-023-03793-6 36750933 PMC9906866

[B21] Lv X. Zhan L. Ye T. Xie H. Chen Z. Lin Y. . (2024). Gut commensal Agathobacter rectalis alleviates microglia-mediated neuroinflammation against pathogenesis of Alzheimer disease. iScience 27:111116. doi: 10.1016/j.isci.2024.111116 39498309 PMC11532950

[B22] Majerníková N. Marmolejo-Garza A. Salinas C. S. Luu M. D. A. Zhang Y. Trombetta-Lima M. . (2024). The link between amyloid β and ferroptosis pathway in Alzheimer's disease progression. Cell Death Dis. 15:782. doi: 10.1038/s41419-024-07152-0 39468028 PMC11519607

[B23] Mohammadi F. Green M. Tolsdorf E. Greffard K. Leclercq M. Bilodeau J. F. . (2023). Industrial and ruminant trans-fatty acids-enriched diets differentially modulate the microbiome and fecal metabolites in C57BL/6 mice. Nutrients 15:1433. doi: 10.3390/nu15061433 36986163 PMC10052023

[B24] Punia S. Juran B. D. Ali A. H. Schlicht E. M. Moore R. M. Sun Z. . (2021). Evaluation of circulating cell-free DNA in cholestatic liver disease using liver-specific methylation markers. BMC Gastroenterol. 21:149. doi: 10.1186/s12876-021-01741-5 33794792 PMC8017778

[B25] Quraishi M. N. Acharjee A. Beggs A. D. Horniblow R. Tselepis C. Gkoutos G. . (2020). A pilot integrative analysis of colonic gene expression, gut microbiota, and immune infiltration in primary sclerosing cholangitis-inflammatory bowel disease: association of disease with bile acid pathways. J. Crohns Colitis 14, 935–947. doi: 10.1093/ecco-jcc/jjaa021 32016358 PMC7392170

[B26] Quraishi M. N. Cheesbrough J. Rimmer P. Mullish B. H. Sharma N. Efstathiou E. . (2025). Open label vancomycin in primary sclerosing cholangitis-inflammatory bowel disease: improved colonic disease activity and associations with changes in host-microbiome-metabolomic signatures. J. Crohns Colitis 19:jjae189. doi: 10.1093/ecco-jcc/jjae189 39673746 PMC11831226

[B27] Ruiz-Álvarez B. E. Padilla-de la Rosa J. D. González Avila M. Sandoval G. Desjardins Y. (2025). Novel acylated naringin enhances propionate release and stimulates the growth of flavanone-metabolizing bacteria in an *in vitro* batch fermentation model. Life (Basel) 15:967. doi: 10.3390/life15060967 40566619 PMC12193867

[B28] Sarkar A. Mitra P. Lahiri A. Das T. Sarkar J. Paul S. . (2023). Butyrate limits inflammatory macrophage niche in NASH. Cell Death Dis. 14:332. doi: 10.1038/s41419-023-05853-6 37202387 PMC10195803

[B29] Shi H. Li H. Yuan R. Guan W. Zhang X. Zhang S. . (2018). PCBP1 depletion promotes tumorigenesis through attenuation of p27Kip1 mRNA stability and translation. J. Exp. Clin. Cancer Res. 37:187. doi: 10.1186/s13046-018-0840-1 30086790 PMC6081911

[B30] Singer M. Valerin J. Zhang Z. Zhang Z. Dayyani F. Yaghmai V. . (2025). Promising cellular immunotherapy for colorectal cancer using classical dendritic cells and natural killer T cells. Cells 14:166. doi: 10.3390/cells14030166 39936958 PMC11817869

[B31] Su M. Tang T. Tang W. Long Y. Wang L. Liu M. (2023). Astragalus improves intestinal barrier function and immunity by acting on intestinal microbiota to treat T2DM: a research review. Front. Immunol. 14:1243834. doi: 10.3389/fimmu.2023.1243834 37638043 PMC10450032

[B32] Tong G. Peng T. Chen Y. Sha L. Dai H. Xiang Y. . (2022). Effects of GLP-1 receptor agonists on biological behavior of colorectal cancer cells by regulating PI3K/AKT/mTOR signaling pathway. Front. Pharmacol. 13:901559. doi: 10.3389/fphar.2022.901559 36034798 PMC9399678

[B33] Toribio-Mateas M. A. Bester A. Klimenko N. (2021). Impact of plant-based meat alternatives on the gut microbiota of consumers: a real-world study. Foods 10:2040. doi: 10.3390/foods10092040 34574149 PMC8465665

[B34] van Munster K. N. Bergquist A. Ponsioen C. Y. (2024). Inflammatory bowel disease and primary sclerosing cholangitis: one disease or two? J. Hepatol. 80, 155–168. doi: 10.1016/j.jhep.2023.09.031 37940453

[B35] Wang X. Yang D. (2021). The regulation of RNA metabolism in hormone signaling and breast cancer. Mol. Cell Endocrinol. 529:111221. doi: 10.1016/j.mce.2021.111221 33711334 PMC8262629

[B36] Wei Y. X. Zheng K. Y. Wang Y. G. (2021). Gut microbiota-derived metabolites as key mucosal barrier modulators in obesity. World J. Gastroenterol. 27, 5555–5565. doi: 10.3748/wjg.v27.i33.5555 34588751 PMC8433617

[B37] Wu T. Li T. Zhang C. Tian Y. Li H. He Y. . (2025). A hollow-tube-like hydrospongel for multimodal therapy of advanced colorectal cancer. Nat. Commun. 16:7464. doi: 10.1038/s41467-025-62880-x 40796777 PMC12343928

[B38] Wu Y. Li Q. Liu J. Liu Y. Xu Y. Zhang R. . (2021). Integrating serum metabolome and gut microbiome to evaluate the benefits of lauric acid on lipopolysaccharide—challenged broilers. Front. Immunol. 12:759323. doi: 10.3389/fimmu.2021.759323 34721434 PMC8554146

[B39] Yang Z. Wang J. Chen Y. Chen T. Shen Z. Wang Y. . (2025). Veillonella intestinal colonization promotes C. difficile infection in Crohn's disease. Cell Host Microbe 33, 1518–1534.e10. doi: 10.1016/j.chom.2025.07.019 40848719

[B40] Yasuda M. Shiokawa M. Kuwada T. Nishikawa Y. Nakanishi R. Takimoto I. . (2025). Anti-integrin αvβ6 autoantibody in primary sclerosing cholangitis: a Japanese nationwide study. J. Gastroenterol. 60, 118–126. doi: 10.1007/s00535-024-02169-w 39549066 PMC11717786

[B41] Yu D. Hu H. Zhang Q. Wang C. Xu M. Xu H. . (2025). Acevaltrate as a novel ferroptosis inducer with dual targets of PCBP1/2 and GPX4 in colorectal cancer. Signal Transduct. Target Ther. 10:211. doi: 10.1038/s41392-025-02296-7 40619432 PMC12230175

[B42] Zhang H. Huang Z. Xue X. Luo X. Guo Z. Miao S. . (2025). Effect of fucoidan molecular weight on gut microbiota composition and anti-inflammatory activity after *in vitro* dynamic digestion and fermentation. *Int. J. Biol. Macromol*. 330(Pt 3):147976. doi: 10.1016/j.ijbiomac.2025.147976 41033509

[B43] Zhang P. Luo Z. Xu Y. Zhang Y. Zhang R. Paerhati N. . (2025). Hypoxia-induced circPRELID2 promotes gastric cancer metastasis by facilitating ZEB2 translation via PCBP1 O-GlcNAcylation. Adv. Sci. (Weinh). 12:e05396. doi: 10.1002/advs.202505396 41117067 PMC12697806

[B44] Zhang Y. Kang Q. He L. Chan K. I. Gu H. Xue W. . (2024). Exploring the immunometabolic potential of Danggui Buxue Decoction for the treatment of IBD-related colorectal cancer. Chin. Med. 19:117. doi: 10.1186/s13020-024-00978-y 39210410 PMC11360867

[B45] Zhu Z. Li M. Weng J. Li S. Guo T. Guo Y. . (2024). LncRNA GAS6-AS1 contributes to 5-fluorouracil resistance in colorectal cancer by facilitating the binding of PCBP1 with MCM3. Cancer Lett. 589:216828. doi: 10.1016/j.canlet.2024.216828 38521199

[B46] Zhuang C. Chen Q. Dou X. Zhang Y. Jin W. Lu X. . (2025). Pathogenic mechanisms of cerebral ischemia and potential gut-brain axis-oriented therapeutic strategies. Phytomedicine 148:157264. doi: 10.1016/j.phymed.2025.157264 40972262

