# Supplementary material for: Gut microbiota pathways linking primary sclerosing cholangitis to colorectal cancer: the Lachnospiraceae family and PCBP1
Source: Front Microbiol. 2026 Apr 24;17:1781475. doi: 10.3389/fmicb.2026.1781475 (PMC13153073; doi:10.3389/fmicb.2026.1781475)
Supplement: Supplementary file 1 [file Data_Sheet_1.zip › Figure Legends(S1-13).docx]

Fig S1. MR leave-one-out sensitivity analysis for *family Rikenellaceae* on colon cancer

Fig S2. MR leave-one-out sensitivity analysis for *genus Adlercreutzia* on colon cancer

Fig S3. MR leave-one-out sensitivity analysis for *genus Allisonella* on colon cancer

Fig S4. MR leave-one-out sensitivity analysis for *genus Blautia* on colon cancer

Fig S5. MR leave-one-out sensitivity analysis for *genus Coprococcus2* on colon cancer

Fig S6. MR leave-one-out sensitivity analysis for *genus Lachnospiraceae* on colon cancer

Fig S7. MR leave-one-out sensitivity analysis for *genus Lactobacillus* on colon cancer

Fig S8. MR leave-one-out sensitivity analysis for colon cancer on *genus Lachnospiraceae*

Fig S9. MR leave-one-out sensitivity analysis for *family Christensenellaceae* on PSC

Fig S10. MR leave-one-out sensitivity analysis for *family Clostridiaceae1* on PSC

Fig S11. MR leave-one-out sensitivity analysis for *family Lachnospiraceae* on PSC

Fig S12. MR leave-one-out sensitivity analysis for *genus Eubacteriumhalliigroup* on PSC

Fig S13. MR leave-one-out sensitivity analysis for *genus Lachnospiraceae* on PSC
