# Supplementary material for: Gut microbiota pathways linking primary sclerosing cholangitis to colorectal cancer: the Lachnospiraceae family and PCBP1
Source: Front Microbiol. 2026 Apr 24;17:1781475. doi: 10.3389/fmicb.2026.1781475 (PMC13153073; doi:10.3389/fmicb.2026.1781475)
Supplement: Supplementary file 1 [file Data_Sheet_1.zip › Table S1.docx]

Table S1. 6 SNPs for the PSC

| POS | OA | EA | SNP | P | BETA | SE | EAF |
| --- | --- | --- | --- | --- | --- | --- | --- |
| 31347597 | C | T | rs9265957 | 6.39E-15 | 0.403259 | 0.0517264 | 0.0943977 |
| 32295681 | T | G | rs6910668 | 1.97E-17 | 0.352283 | 0.0414663 | 0.172016 |
| 32626411 | A | G | rs9271768 | 1.55E-10 | 0.230375 | 0.0359939 | 0.296094 |
| 32724958 | A | T | rs9275924 | 5.48E-15 | 0.311111 | 0.039807 | 0.199568 |
| 33831708 | C | T | rs62397578 | 9.57E-07 | 0.290084 | 0.0591966 | 0.0763561 |
| 39891891 | T | C | rs7760703 | 9.04E-07 | -0.346087 | 0.0704653 | 0.0792489 |

Abbreviation: single-nucleotide polymorphism (SNP); effect allele (EA); other allele (OA); effect sizes (BETA);

standard error (SE); Effect Allele Frequency (EAF)
