# Supplementary material for: Gut microbiota pathways linking primary sclerosing cholangitis to colorectal cancer: the Lachnospiraceae family and PCBP1
Source: Front Microbiol. 2026 Apr 24;17:1781475. doi: 10.3389/fmicb.2026.1781475 (PMC13153073; doi:10.3389/fmicb.2026.1781475)
Supplement: Supplementary file 1 [file Data_Sheet_1.zip › Table S2.docx]

Table S2. 89 SNPs for seven gut microbiotas

| BAC | CHR | POS | SNP | OA | EA | BETA | SE | EAF | P |
| --- | --- | --- | --- | --- | --- | --- | --- | --- | --- |
| family.Rikenellaceae. | 15 | 71658642 | rs4264350 | C | T | -0.05264 | 0.010855489 | -4.832495415 | 1.35E-06 |
| family.Rikenellaceae. | 9 | 14158854 | rs62532512 | A | C | -0.05043 | 0.010739924 | -4.687601419 | 2.76E-06 |
| family.Rikenellaceae. | 7 | 135778919 | rs67281112 | C | G | 0.063581 | 0.013745123 | 4.631243976 | 3.63E-06 |
| family.Rikenellaceae. | 4 | 187842232 | rs6837275 | G | A | 0.057035 | 0.011886994 | 4.818419461 | 1.45E-06 |
| family.Rikenellaceae. | 13 | 38044689 | rs9603208 | T | G | 0.082117 | 0.015959564 | 5.2071129 | 1.92E-07 |
| family.Rikenellaceae. | 1 | 19790706 | rs67705352 | G | T | -0.05497 | 0.01105338 | -4.973497615 | 6.58E-07 |
| family.Rikenellaceae. | 11 | 95304834 | rs1939881 | A | G | -0.10559 | 0.020621272 | -5.003025942 | 5.64E-07 |
| family.Rikenellaceae. | 13 | 22867356 | rs9578457 | A | G | -0.14148 | 0.031584793 | -4.611669087 | 3.99E-06 |
| family.Rikenellaceae. | 21 | 32496710 | rs2833282 | A | G | 0.071114 | 0.015696614 | 4.595793459 | 4.31E-06 |
| family.Rikenellaceae. | 4 | 12159480 | rs4306975 | G | T | 0.053296 | 0.011735734 | 4.513949706 | 6.36E-06 |
| family.Rikenellaceae. | 14 | 21548634 | rs77885767 | T | C | -0.15621 | 0.033648181 | -4.681564046 | 2.85E-06 |
| family.Rikenellaceae. | 18 | 4069710 | rs59663348 | A | G | 0.057091 | 0.012504754 | 4.522116367 | 6.12E-06 |
| family.Rikenellaceae. | 14 | 90270821 | rs74474130 | G | T | 0.137747 | 0.029568753 | 4.632441802 | 3.61E-06 |
| family.Rikenellaceae. | 18 | 10338470 | rs7242694 | T | C | -0.06187 | 0.013488231 | -4.533293834 | 5.81E-06 |
| family.Rikenellaceae. | 21 | 28515912 | rs36021379 | G | A | -0.0656 | 0.014490275 | -4.487830875 | 7.20E-06 |
| family.Rikenellaceae. | 9 | 86052655 | rs10217435 | T | C | -0.08838 | 0.019750648 | -4.509188767 | 6.51E-06 |
| family.Rikenellaceae. | 8 | 99188924 | rs2447496 | A | G | -0.05492 | 0.012190433 | -4.523376057 | 6.09E-06 |
| family.Rikenellaceae. | 8 | 144441740 | rs35909684 | C | A | -0.08502 | 0.01915389 | -4.490720707 | 7.10E-06 |
| family.Rikenellaceae. | 16 | 85396629 | rs4783173 | G | C | 0.048151 | 0.011000589 | 4.491747896 | 7.06E-06 |
| family.Rikenellaceae. | 11 | 17589129 | rs10832801 | C | A | -0.05348 | 0.012266628 | -4.478862947 | 7.50E-06 |
| family.Rikenellaceae. | 6 | 100244667 | rs9389714 | T | C | -0.06368 | 0.014341562 | -4.444964066 | 8.79E-06 |
| family.Rikenellaceae. | 2 | 174257127 | rs6744030 | T | C | 0.069675 | 0.015710296 | 4.432489186 | 9.32E-06 |
| genus.Adlercreutzia. | 4 | 171281359 | rs7680684 | T | C | -0.08338 | 0.016890088 | -4.896234666 | 9.77E-07 |
| genus.Adlercreutzia. | 1 | 201061818 | rs2147798 | G | C | 0.092296 | 0.019212124 | 4.82517954 | 1.40E-06 |
| genus.Adlercreutzia. | 5 | 94059857 | rs80078995 | T | A | -0.11324 | 0.023258622 | -4.801656596 | 1.57E-06 |
| genus.Adlercreutzia. | 18 | 75009770 | rs2717140 | T | C | -0.11923 | 0.025108114 | -4.748832666 | 2.05E-06 |
| genus.Adlercreutzia. | 6 | 123909083 | rs9490822 | T | C | -0.07345 | 0.015578896 | -4.70455471 | 2.54E-06 |
| genus.Adlercreutzia. | 1 | 69444163 | rs6664405 | C | T | -0.09531 | 0.02107517 | -4.555174309 | 5.23E-06 |
| genus.Adlercreutzia. | 5 | 31991834 | rs12522517 | T | A | -0.10488 | 0.023469475 | -4.590940166 | 4.41E-06 |
| genus.Adlercreutzia. | 7 | 48844151 | rs13231526 | A | C | 0.143237 | 0.031165443 | 4.57275415 | 4.81E-06 |
| genus.Adlercreutzia. | 10 | 135204950 | rs1046175 | G | C | 0.112758 | 0.025599407 | 4.514091038 | 6.36E-06 |
| genus.Adlercreutzia. | 3 | 105355971 | rs55719207 | A | G | -0.06992 | 0.015803609 | -4.425878781 | 9.61E-06 |
| genus.Adlercreutzia. | 17 | 48864179 | rs9915817 | C | T | 0.074922 | 0.016833007 | 4.459457661 | 8.22E-06 |
| genus.Adlercreutzia. | 11 | 98386085 | rs11604400 | T | C | -0.10252 | 0.023483368 | -4.422763724 | 9.74E-06 |
| genus.Allisonella. | 9 | 79110160 | rs602075 | G | A | 0.168974 | 0.029697566 | 5.511111432 | 3.57E-08 |
| genus.Allisonella. | 2 | 33607071 | rs6742198 | A | G | 0.149152 | 0.031648438 | 4.648403361 | 3.35E-06 |
| genus.Allisonella. | 9 | 100150556 | rs35778461 | T | C | 0.146679 | 0.029718557 | 4.85460075 | 1.21E-06 |
| genus.Allisonella. | 5 | 114286707 | rs1901739 | G | T | 0.115769 | 0.024862618 | 4.633922673 | 3.59E-06 |
| genus.Allisonella. | 7 | 137968010 | rs76904847 | A | G | 0.148523 | 0.033485691 | 4.52332694 | 6.09E-06 |
| genus.Allisonella. | 12 | 28018998 | rs35110698 | C | T | -0.14632 | 0.032085207 | -4.53636452 | 5.72E-06 |
| genus.Allisonella. | 10 | 122413451 | rs7898615 | G | T | 0.167966 | 0.037358631 | 4.442964827 | 8.87E-06 |
| genus.Allisonella. | 11 | 88632972 | rs594561 | T | C | 0.112231 | 0.025168069 | 4.430281151 | 9.41E-06 |
| genus.Allisonella. | 18 | 75370866 | rs685403 | C | G | -0.1752 | 0.040447416 | -4.569712386 | 4.88E-06 |
| genus.Blautia. | 16 | 77238810 | rs11149971 | T | C | 0.117605 | 0.023389975 | 4.884040877 | 1.04E-06 |
| genus.Blautia. | 17 | 16895873 | rs12453000 | T | C | 0.062533 | 0.012998711 | 4.845777301 | 1.26E-06 |
| genus.Blautia. | 1 | 175757311 | rs115043014 | A | G | -0.20661 | 0.043991974 | -4.557119378 | 5.19E-06 |
| genus.Blautia. | 7 | 124210039 | rs67794373 | T | C | 0.060171 | 0.012344281 | 4.891632696 | 1.00E-06 |
| genus.Blautia. | 7 | 68396262 | rs117001700 | C | T | 0.196414 | 0.044112197 | 4.443781902 | 8.84E-06 |
| genus.Blautia. | 19 | 1043103 | rs72973581 | G | A | 0.125169 | 0.026540793 | 4.781567394 | 1.74E-06 |
| genus.Blautia. | 19 | 13420917 | rs4926264 | C | T | 0.082621 | 0.017823907 | 4.560510934 | 5.10E-06 |
| genus.Blautia. | 9 | 1866537 | rs7860714 | G | A | -0.05022 | 0.010985269 | -4.606680852 | 4.09E-06 |
| genus.Blautia. | 11 | 87805911 | rs682885 | G | A | -0.04934 | 0.010735883 | -4.587270057 | 4.49E-06 |
| genus.Blautia. | 1 | 219117743 | rs2788271 | G | T | -0.05756 | 0.013349169 | -4.488760326 | 7.16E-06 |
| genus.Blautia. | 14 | 78062644 | rs113271346 | T | C | 0.078268 | 0.017211647 | 4.498176944 | 6.85E-06 |
| genus.Blautia. | 6 | 74605806 | rs3005511 | G | A | 0.050108 | 0.01107676 | 4.51982588 | 6.19E-06 |
| genus.Blautia. | 6 | 9298640 | rs16892041 | C | T | -0.06226 | 0.014180398 | -4.444240636 | 8.82E-06 |
| genus.Coprococcus2. | 3 | 180544880 | rs12634070 | C | T | 0.073649 | 0.016492134 | 4.418234597 | 9.95E-06 |
| genus.Coprococcus2. | 1 | 223688236 | rs61823518 | C | A | -0.09554 | 0.021572431 | -4.503555947 | 6.68E-06 |
| genus.Coprococcus2. | 5 | 34794789 | rs10070053 | G | A | 0.059431 | 0.01353752 | 4.474784923 | 7.65E-06 |
| genus.Coprococcus2. | 9 | 25554068 | rs2482516 | T | C | 0.075441 | 0.016461865 | 4.576715374 | 4.72E-06 |
| genus.Coprococcus2. | 20 | 49266770 | rs59936925 | T | A | 0.117085 | 0.023399473 | 4.904313748 | 9.38E-07 |
| genus.Coprococcus2. | 1 | 112139008 | rs6677933 | T | C | -0.08044 | 0.016421626 | -4.858126895 | 1.19E-06 |
| genus.Coprococcus2. | 14 | 86833255 | rs1958519 | A | T | 0.066529 | 0.013855806 | 4.801232369 | 1.58E-06 |
| genus.Coprococcus2. | 4 | 131125786 | rs72680320 | C | T | -0.06494 | 0.013918999 | -4.727666637 | 2.27E-06 |
| genus.Coprococcus2. | 10 | 129715759 | rs35890118 | G | A | -0.06654 | 0.014766106 | -4.458341592 | 8.26E-06 |
| genus.Coprococcus2. | 1 | 4210455 | rs9426473 | G | A | 0.072736 | 0.016166159 | 4.515831925 | 6.31E-06 |
| genus.LachnospiraceaeFCS020group. | 11 | 3248876 | rs35035870 | C | T | -0.19062 | 0.041440269 | -4.698539696 | 2.62E-06 |
| genus.LachnospiraceaeFCS020group. | 3 | 165996415 | rs2862811 | C | T | 0.056493 | 0.012173604 | 4.615626599 | 3.92E-06 |
| genus.LachnospiraceaeFCS020group. | 10 | 44346094 | rs1254846 | A | G | 0.105963 | 0.023250118 | 4.541081592 | 5.60E-06 |
| genus.LachnospiraceaeFCS020group. | 6 | 71966268 | rs4452603 | G | T | 0.060421 | 0.013596453 | 4.440334783 | 8.98E-06 |
| genus.LachnospiraceaeFCS020group. | 4 | 166588085 | rs2322265 | T | C | -0.06663 | 0.014157654 | -4.556226164 | 5.21E-06 |
| genus.LachnospiraceaeFCS020group. | 10 | 65563647 | rs3999074 | T | G | -0.05506 | 0.012184677 | -4.507795194 | 6.55E-06 |
| genus.LachnospiraceaeFCS020group. | 8 | 121244406 | rs10093861 | A | G | -0.05689 | 0.012115188 | -4.667003415 | 3.06E-06 |
| genus.LachnospiraceaeFCS020group. | 19 | 2571232 | rs7249113 | A | G | 0.067948 | 0.013349587 | 5.08270544 | 3.72E-07 |
| genus.LachnospiraceaeFCS020group. | 1 | 35328576 | rs12078956 | G | C | 0.106101 | 0.022290421 | 4.738382735 | 2.15E-06 |
| genus.LachnospiraceaeFCS020group. | 16 | 23631307 | rs369444 | G | C | 0.125481 | 0.025900786 | 4.660902367 | 3.15E-06 |
| genus.LachnospiraceaeFCS020group. | 2 | 53946431 | rs72793667 | G | A | -0.11688 | 0.024658906 | -4.794082291 | 1.63E-06 |
| genus.LachnospiraceaeFCS020group. | 19 | 17865305 | rs1363769 | C | T | -0.20063 | 0.044937217 | -4.800635392 | 1.58E-06 |
| genus.LachnospiraceaeFCS020group. | 5 | 109403564 | rs113859143 | C | G | -0.10891 | 0.024205472 | -4.70373063 | 2.55E-06 |
| genus.LachnospiraceaeFCS020group. | 13 | 40419314 | rs9788306 | T | C | -0.0628 | 0.013074422 | -4.826981479 | 1.39E-06 |
| genus.LachnospiraceaeFCS020group. | 1 | 118425334 | rs9919338 | C | G | -0.05533 | 0.012062109 | -4.568739198 | 4.91E-06 |
| genus.LachnospiraceaeFCS020group. | 4 | 165786478 | rs9308097 | G | A | 0.055381 | 0.012365944 | 4.479754313 | 7.47E-06 |
| genus.Lactobacillus. | 19 | 6928017 | rs921925 | C | A | 0.098508 | 0.020322897 | 4.897291713 | 9.72E-07 |
| genus.Lactobacillus. | 1 | 18501459 | rs16861661 | A | G | -0.18315 | 0.038148013 | -4.843130105 | 1.28E-06 |
| genus.Lactobacillus. | 8 | 69010103 | rs768253 | G | T | -0.0792 | 0.017179101 | -4.59887935 | 4.25E-06 |
| genus.Lactobacillus. | 2 | 28903275 | rs11674854 | T | C | -0.08527 | 0.017650364 | -4.799326519 | 1.59E-06 |
| genus.Lactobacillus. | 16 | 73890910 | rs328312 | A | T | 0.081509 | 0.016944121 | 4.824126457 | 1.41E-06 |
| genus.Lactobacillus. | 20 | 54192992 | rs6092149 | T | A | -0.08013 | 0.01714896 | -4.651805029 | 3.29E-06 |
| genus.Lactobacillus. | 6 | 151027636 | rs75127669 | A | C | 0.139784 | 0.031041446 | 4.498783621 | 6.83E-06 |
| genus.Lactobacillus. | 3 | 173151665 | rs77478751 | G | A | -0.21989 | 0.047575911 | -4.483959582 | 7.33E-06 |
| genus.Lactobacillus. | 2 | 135755629 | rs1530559 | A | G | 0.0804 | 0.017820739 | 4.567698211 | 4.93E-06 |
| genus.Lactobacillus. | 4 | 109896462 | rs62314653 | A | C | 0.187692 | 0.039458456 | 4.7301235 | 2.24E-06 |
| genus.Lactobacillus. | 13 | 23834968 | rs7399658 | A | G | -0.10713 | 0.022188263 | -4.662692289 | 3.12E-06 |
| genus.Lactobacillus. | 2 | 199312684 | rs12693845 | T | C | -0.08054 | 0.017742876 | -4.440783337 | 8.96E-06 |

Abbreviation: single-nucleotide polymorphism (SNP); effect allele (EA); other allele (OA); effect sizes (BETA); standard error (SE); Effect Allele Frequency (EAF)
