# Supplementary material for: Gut microbiota pathways linking primary sclerosing cholangitis to colorectal cancer: the Lachnospiraceae family and PCBP1
Source: Front Microbiol. 2026 Apr 24;17:1781475. doi: 10.3389/fmicb.2026.1781475 (PMC13153073; doi:10.3389/fmicb.2026.1781475)
Supplement: Supplementary file 1 [file Data_Sheet_1.zip › Table S3.docx]

Table S3. Cochran's Q test and Pleiotropy test

| Exposure | | Outcome | | nSNP | OR(95% CI) | P | Cochran's Q test | Pleiotropy test |
| --- | --- | --- | --- | --- | --- | --- | --- | --- |
| PSC | | Colon Cancer | | 6 | 1.172(1.032-1.332) | 0.014 | 0.606 | 0.587 |
| PSC | | Gut microbiota | C.Methanobacteria | 2 | 0.835(0.710-0.983) | 0.03 | *NA* | *NA* |
|  |  |  | F.Christensenellaceae | 3 | 0.924(0.862-0.990) | 0.026 | 0.505 | 0.97 |
|  |  |  | F.Methanobacteriaceae | 2 | 0.835(0.710-0.983) | 0.03 | *NA* | *NA* |
|  |  |  | F.Oxalobacteraceae | 2 | 0.866(0.758-0.990) | 0.035 | *NA* | *NA* |
|  |  |  | F.Rhodospirillaceae | 2 | 1.113(1.002-1.235) | 0.044 | *NA* | *NA* |
|  |  |  | F.Streptococcaceae | 3 | 1.092(1.009-1.182) | 0.027 | 0.276 | 0.581 |
|  |  |  | G.Anaerofilum | 2 | 1.161(1.016-1.328) | 0.028 | *NA* | *NA* |
|  |  |  | G.Christensenellaceae | 3 | 0.918(0.855-0.984) | 0.017 | 0.67 | 0.756 |
|  |  |  | G.Lachnospira | 3 | 1.092(1.018-1.173) | 0.013 | 0.359 | 0.389 |
|  |  |  | G.Lachnospiraceae FCS020 | 3 | 1.108(1.030-1.191) | 0.005 | 0.468 | 0.753 |
|  |  |  | G.Oxalobacter | 2 | 0.844(0.733-0.972) | 0.018 | *NA* | *NA* |
|  |  |  | G.Streptococcus | 3 | 1.097(1.022-1.176) | 0.009 | 0.397 | 0.488 |
|  |  |  | O.Methanobacteriales | 2 | 0.835(0.710-0.983) | 0.03 | *NA* | *NA* |
|  |  |  | P.Euryarchaeota | 2 | 0.825(0.703-0.967) | 0.018 | *NA* | *NA* |
|  |  |  | P.Firmicutes | 3 | 1.081(1.011-1.155) | 0.021 | 0.807 | 0.719 |
| Gut microbiota | F.Rikenellaceae | Colon Cancer | | 19 | 1.243(1.001-1.542) | 0.048 | 0.731 | 0.565 |
|  | G.Adlercreutzia |  |  | 11 | 1.350(1.053-1.732) | 0.017 | 0.109 | 0.61 |
|  | G.Allisonella |  |  | 9 | 1.175(1.026-1.345) | 0.019 | 0.79 | 0.571 |
|  | G.Blautia |  |  | 13 | 1.352(1.038-1.761) | 0.025 | 0.383 | 0.151 |
|  | G.Coprococcus2 |  |  | 10 | 0.655(0.520-0.827) | 0.0003 | 0.814 | 0.423 |
|  | G.Lachnospiraceae FCS020 |  |  | 15 | 1.237(1.004-1.523) | 0.045 | 0.315 | 0.073 |
|  | G.Lactobacillus |  |  | 12 | 1.199(1.010-1.422) | 0.01 | 0.938 | 0.839 |
| Colon Cancer | | PSC | | 5 | 0.952(0.716-1.267) | 0.739* | 0.142 | 0.121 |
|  |  | Gut microbiota | G.Lachnospiraceae | 4 | 0.914(0.845-0.989) | 0.026 | 0.391 | 0.764 |
| Gut microbiota | F.Christensenellaceae | PSC | | 11 | 0.724(0.532-0.985) | 0.039 | 0.882 | 0.791 |
|  | F.Clostridiaceae1 |  |  | 11 | 0.665(0.457-0.969) | 0.033 | 0.352 | 0.458 |
|  | F.Lachnospiraceae |  |  | 16 | 0.633(0.410-0.977) | 0.039 | 0.92 | 0.764 |
|  | G.Eubacterium hallii |  |  | 13 | 1.665(1.197-2.316) | 0.002 | 0.915 | 0.804 |
|  | G.Lachnospiraceae UCG008 |  |  | 11 | 0.780(0.611-0.996) | 0.046 | 0.67 | 0.174 |
