# Supplementary figures and images for: Gut microbiota pathways linking primary sclerosing cholangitis to colorectal cancer: the Lachnospiraceae family and PCBP1
Source: Front Microbiol. 2026 Apr 24;17:1781475. doi: 10.3389/fmicb.2026.1781475 (PMC13153073; doi:10.3389/fmicb.2026.1781475)

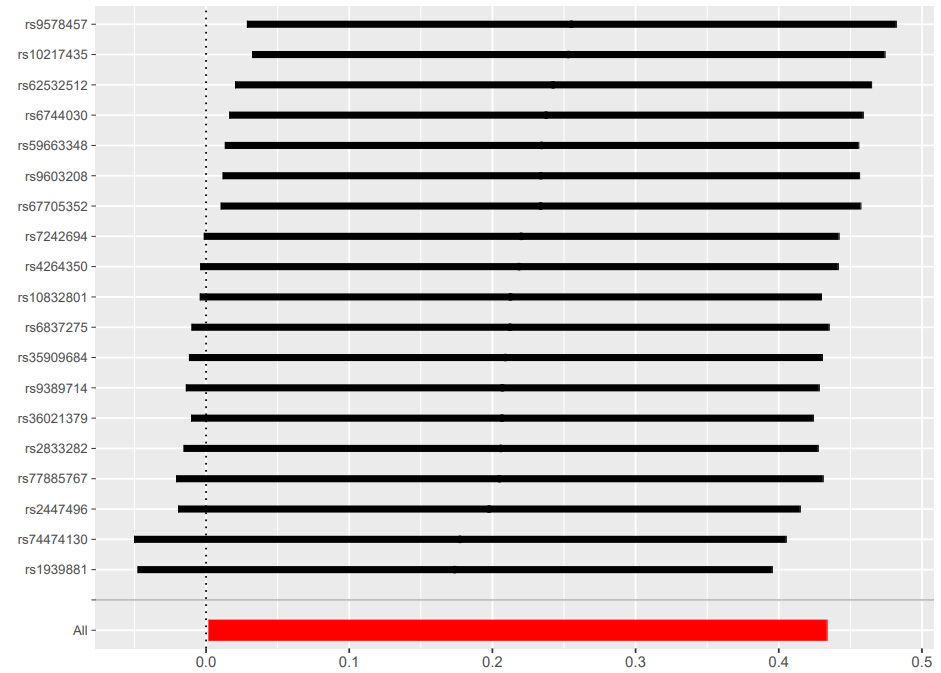

Supplement: Supplementary file 1 [file Data_Sheet_1.zip › Fig S1.jpg]

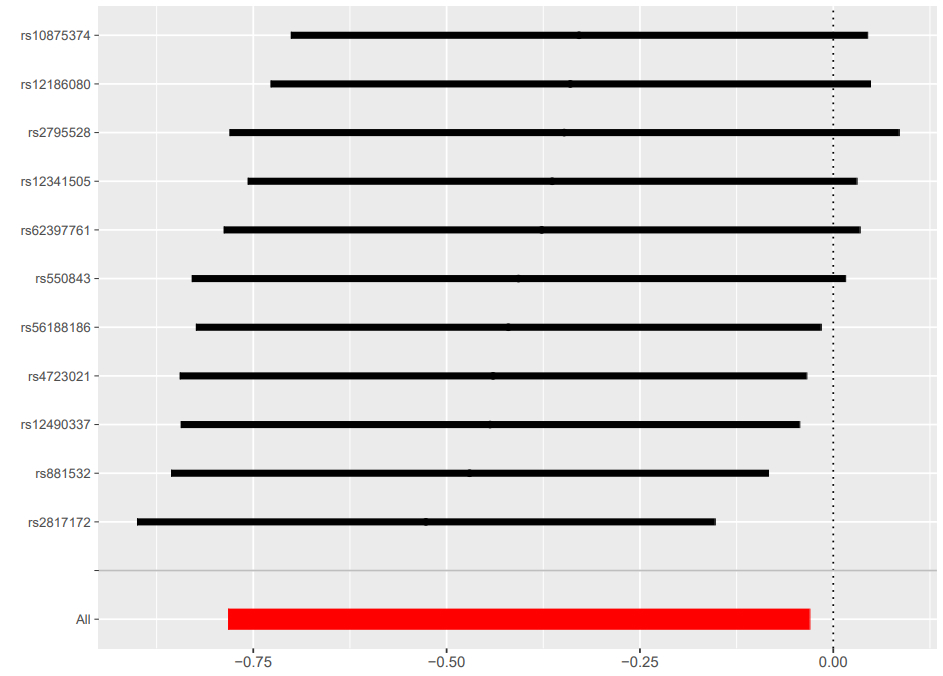

Supplement: Supplementary file 1 [file Data_Sheet_1.zip › Fig S10.jpg]

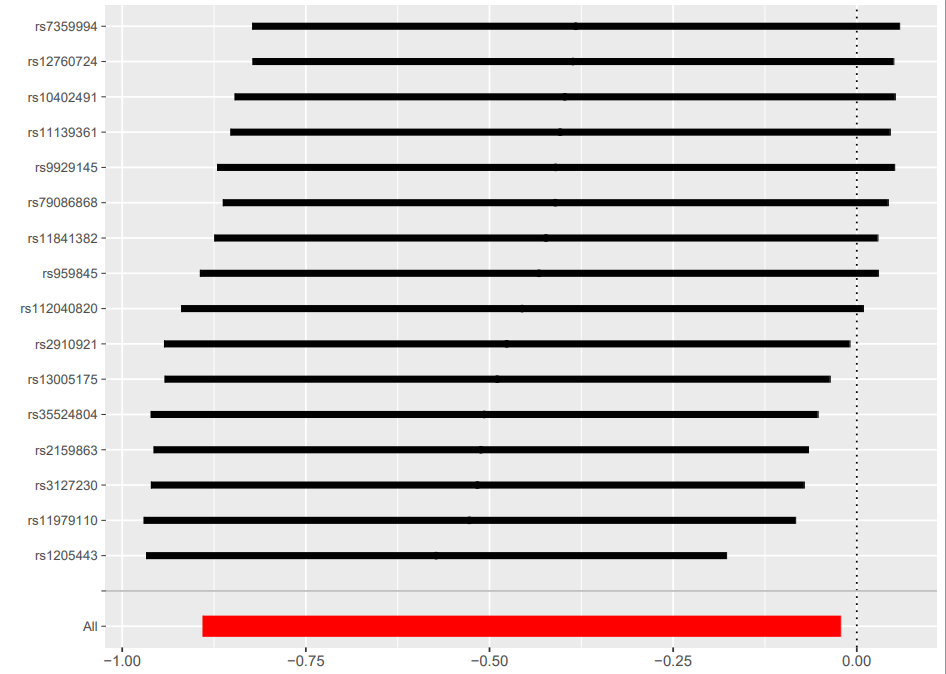

Supplement: Supplementary file 1 [file Data_Sheet_1.zip › Fig S11.jpg]

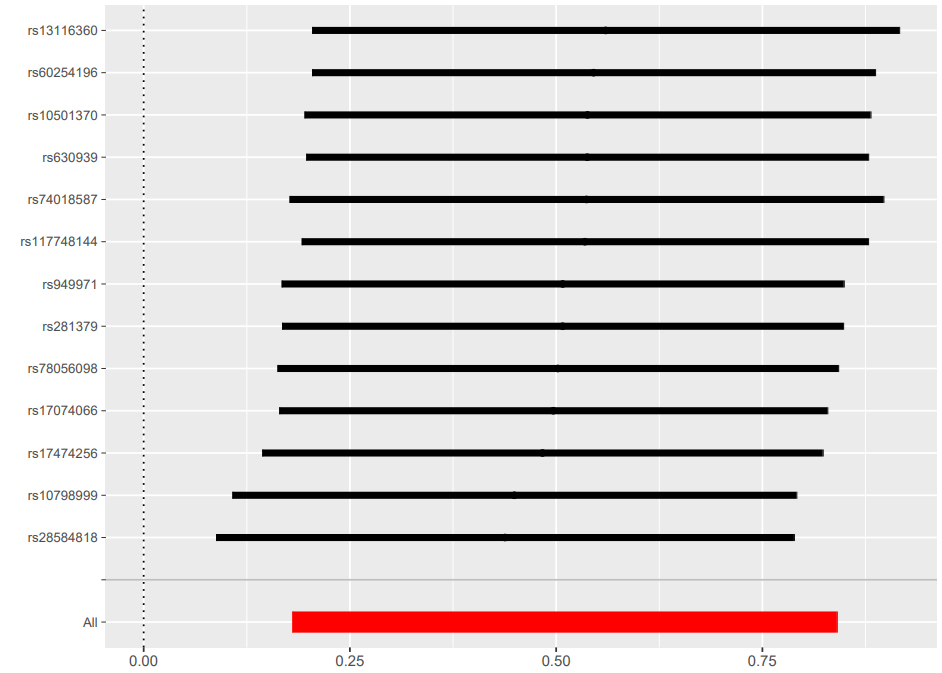

Supplement: Supplementary file 1 [file Data_Sheet_1.zip › Fig S12.jpg]

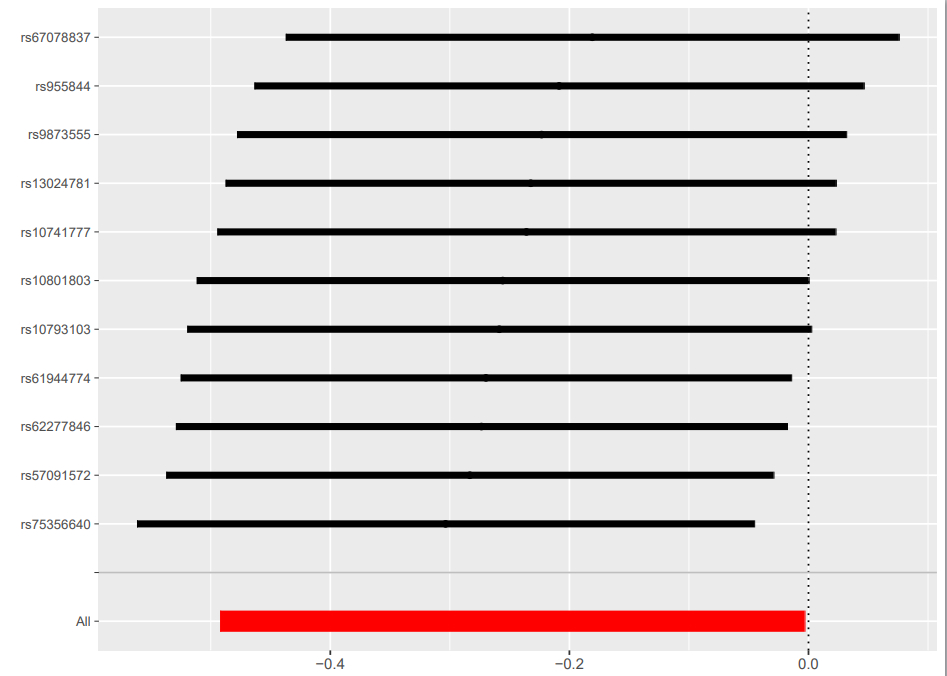

Supplement: Supplementary file 1 [file Data_Sheet_1.zip › Fig S13.jpg]

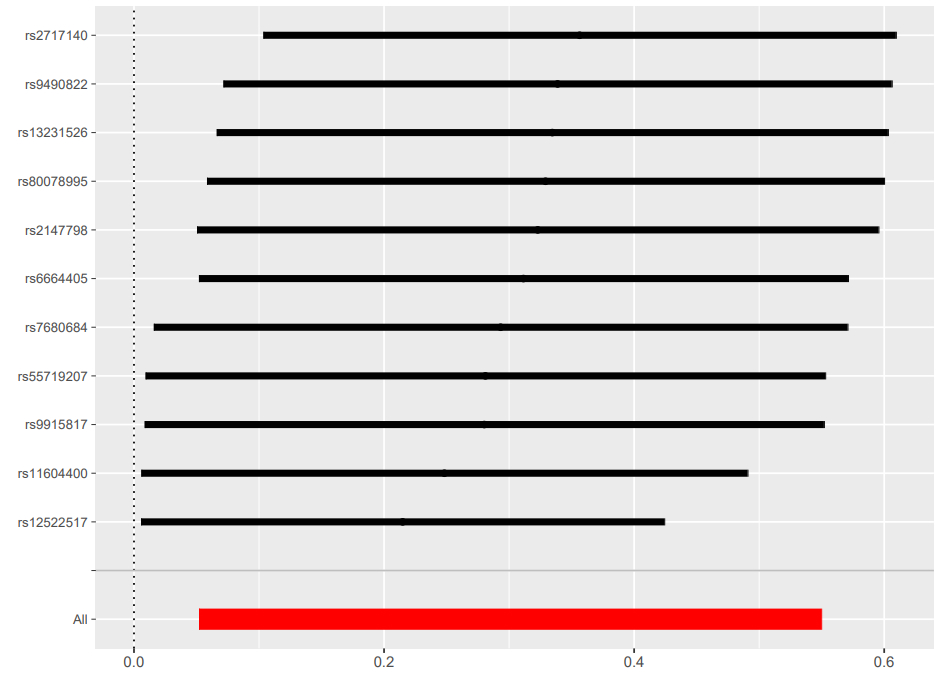

Supplement: Supplementary file 1 [file Data_Sheet_1.zip › Fig S2.jpg]

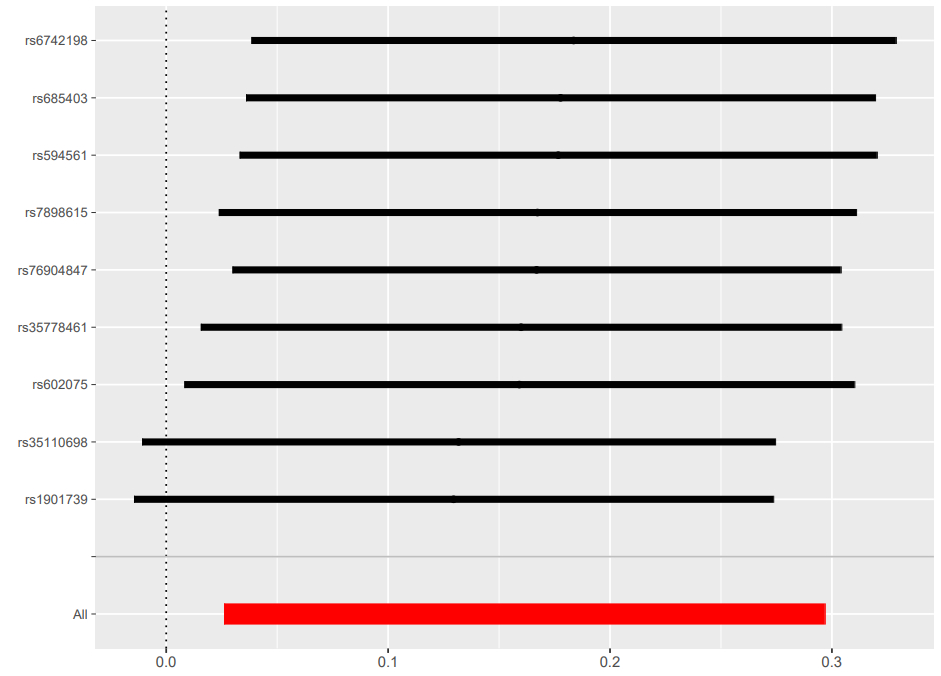

Supplement: Supplementary file 1 [file Data_Sheet_1.zip › Fig S3.jpg]

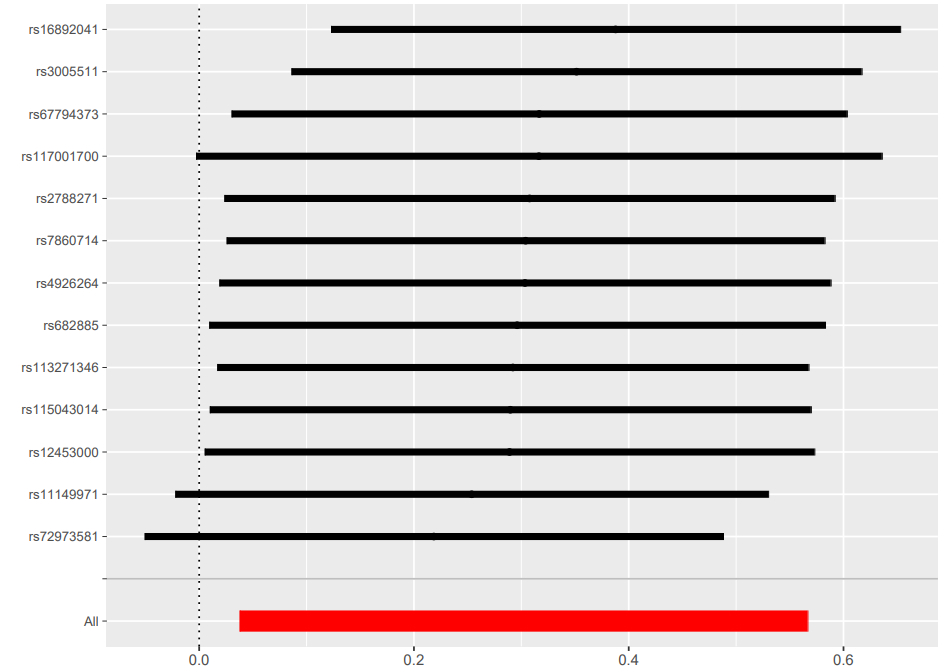

Supplement: Supplementary file 1 [file Data_Sheet_1.zip › Fig S4.jpg]

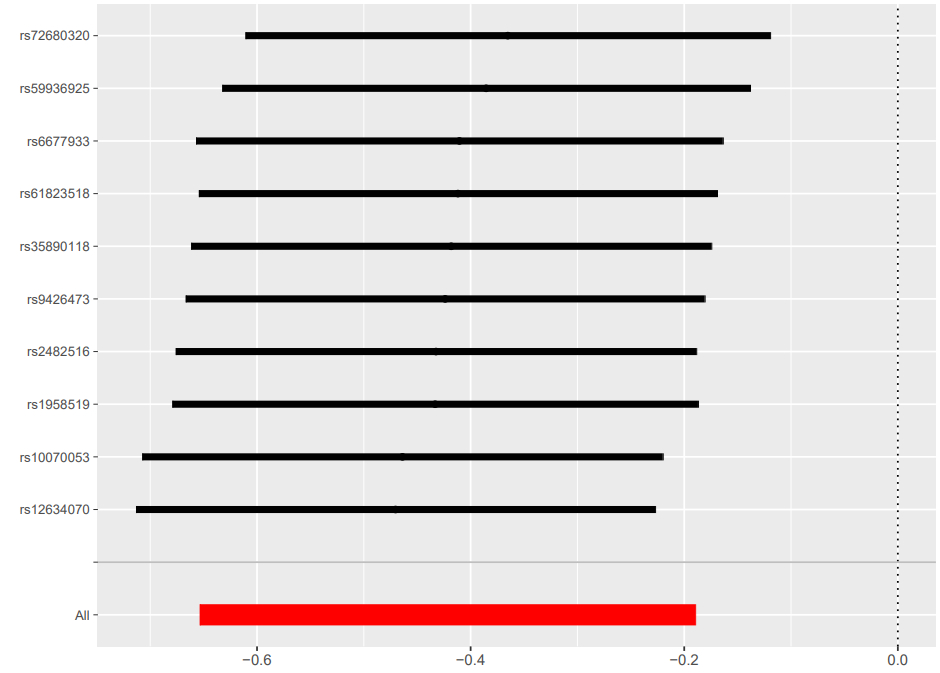

Supplement: Supplementary file 1 [file Data_Sheet_1.zip › Fig S5.jpg]

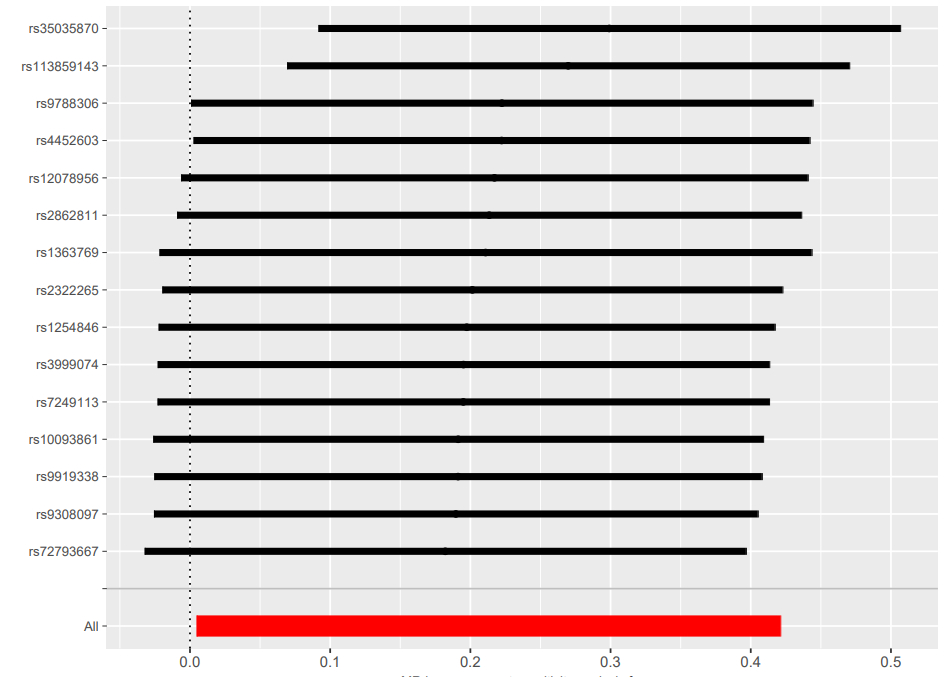

Supplement: Supplementary file 1 [file Data_Sheet_1.zip › Fig S6.jpg]

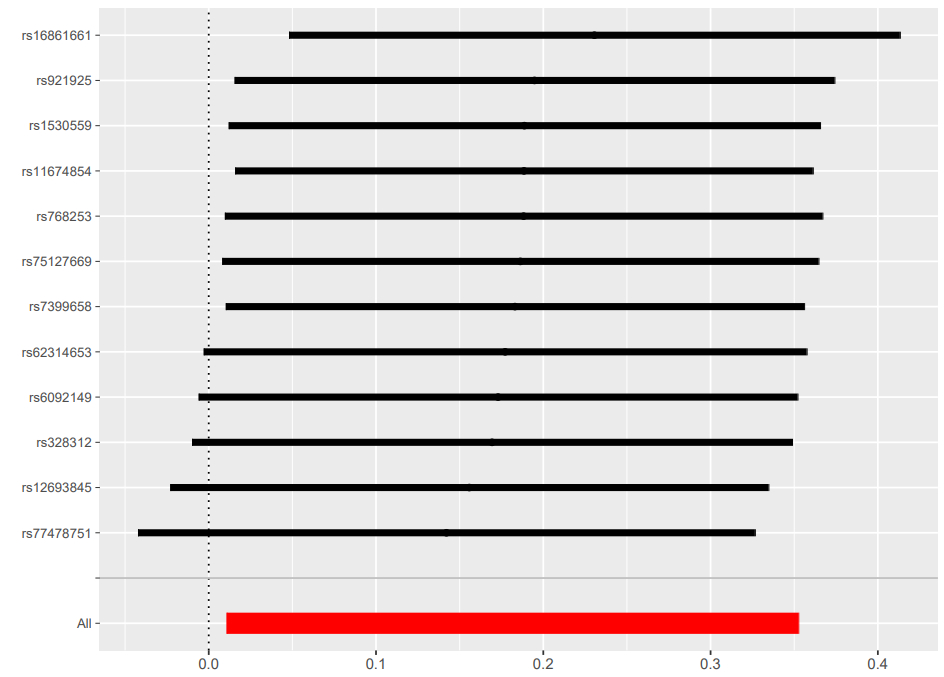

Supplement: Supplementary file 1 [file Data_Sheet_1.zip › Fig S7.jpg]

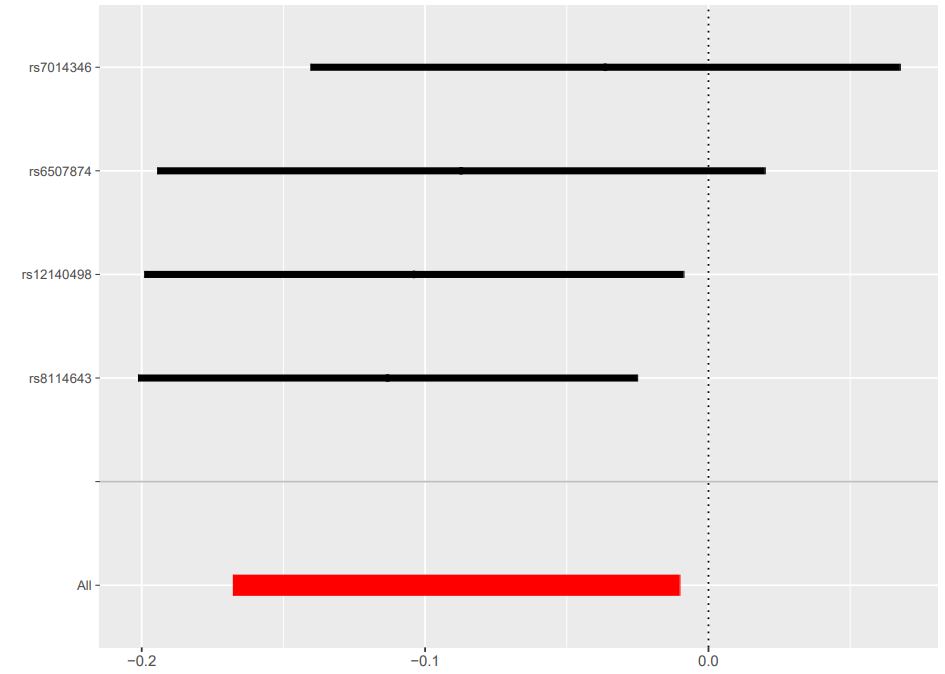

Supplement: Supplementary file 1 [file Data_Sheet_1.zip › Fig S8.jpg]

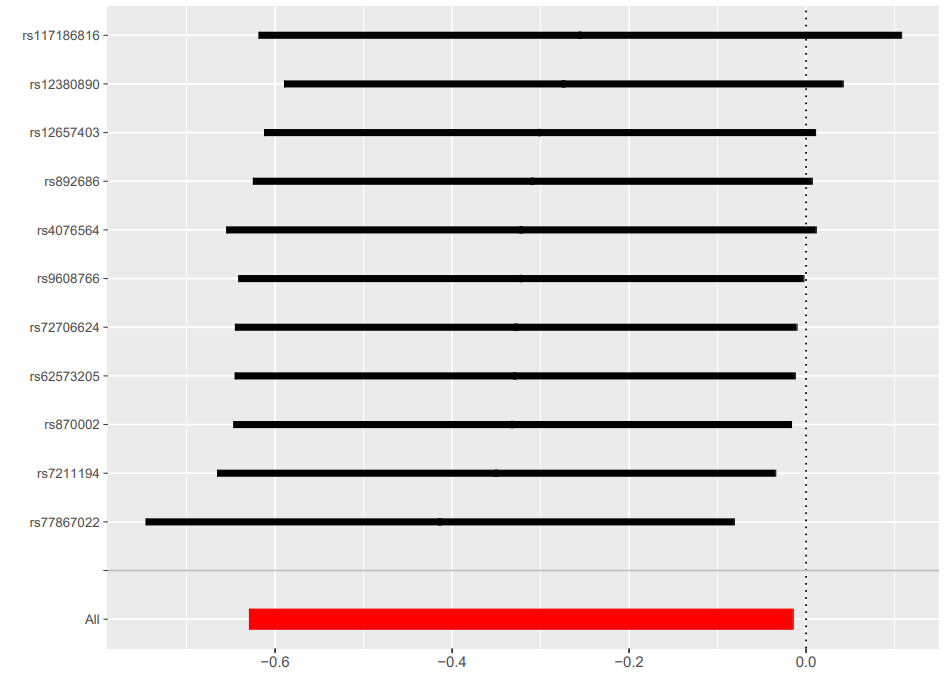

Supplement: Supplementary file 1 [file Data_Sheet_1.zip › Fig S9.jpg]
